# BRAKER3: Fully Automated Genome Annotation Using RNA-Seq and Protein Evidence with GeneMark-ETP, AUGUSTUS and TSEBRA

**DOI:** 10.1101/2023.06.10.544449

**Published:** 2023-09-02

**Authors:** Lars Gabriel, Tomáš Brůna, Katharina J. Hoff, Matthis Ebel, Alexandre Lomsadze, Mark Borodovsky, Mario Stanke

**Affiliations:** 1 Institute of Mathematics and Computer Science, University of Greifswald, 17489 Greifswald, Germany; 2 Center for Functional Genomics of Microbes, University of Greifswald, 17489 Greifswald, Germany; 3 Wallace H. Coulter Department of Biomedical Engineering, Georgia Institute of Technology, Atlanta, GA 30332, USA; 4 School of Computational Science and Engineering, Georgia Institute of Technology, Atlanta, GA 30332, USA; 5 U.S. Department of Energy, Joint Genome Institute, Lawrence Berkeley National Laboratory, Berkeley, CA 94720, USA

## Abstract

Gene prediction remains an active area of bioinformatics research. Challenges are presented by large eukaryotic genomes and heterogeneous data situations. To meet the challenges, several streams of evidence must be integrated, from protein homology and transcriptome data, as well as information derived from the genome itself. The amount and significance of the available evidence from transcriptomes and proteomes vary from genome to genome, between genes and even along a single gene. User-friendly and accurate annotation pipelines that can cope with such data heterogeneity are needed. The previously developed annotation pipelines BRAKER1 and BRAKER2 use RNA-Seq or protein data, respectively, but not both. The recently released GeneMark-ETP integrates all three types of data and achieves much higher levels of accuracy.

We here present the BRAKER3 pipeline that builds on GeneMark-ETP and AUGUSTUS and further improves accuracy using the TSEBRA combiner. BRAKER3 annotates protein-coding genes in eukaryotic genomes using both short-read RNA-Seq and a large protein database along with statistical models learned iteratively and specifically for the target genome. We benchmarked the new pipeline on 11 species under controlled conditions on the assumed relatedness of the target species to available proteomes. BRAKER3 outperformed BRAKER1 and BRAKER2, increasing the average transcript-level F1-score by ~20 percentage points, most pronounced for species with large and complex genomes. BRAKER3 also outperforms MAKER2 and Funannotate. For the first time, we provide a Singularity container for the BRAKER software to minimize installation obstacles. Overall, BRAKER3 is an accurate, easy-to-use tool for the annotation of eukaryotic genomes.

## Introduction

New eukaryotic genomes are being sequenced at increasing rates. However, the pace of genome annotation, which establishes links between genomic sequence and biological function, is lagging behind. For example, in April 2023 49% of the eukaryotic species with assemblies in GenBank, had no annotation in GenBank. Undertakings such as the Earth BioGenome Project (https://www.earthbiogenome.org), which aims to annotate ~1.5 million eukaryotic species, further require that the annotation pipeline is highly automated and reliable and ideally no manual work for each species is required when genome assembly and RNA-Seq are given.

Further, species which have an annotation also require re-annotation as assemblies improve or the available extrinsic evidence increases substantially [NCBI, 2023]. This demand further increases the importance of the availability of fast and accurate genome annotation tools.

Current state-of-the-art annotation pipelines integrate extrinsic and intrinsic evidence. Extrinsic evidence is extracted from transcripts and cross-species homologous proteins. RNA-Seq reads offer direct evidence on introns and, if assembled, on a gene structure. Protein sequences from related genomes can be used to identify regions of a genome that encode proteins with similar sequences to known proteins. Due to the sequence divergence between informant and target gene, this evidence may be less reliable and less precise than the one from (native) RNA-Seq alignments. The availability of extrinsic evidence is increasing rapidly. Second-generation sequencing technology has become cheap [Kris A. Wetterstrand, 2021] and RNA-Seq often accompanies genome sequencing. To give an example for protein database growth, OrthoDB’s latest release (v11) includes more than 50% additional eukaryotic species compared to its previous version [Kuznetsov et al., 2023].

Despite the importance of extrinsic evidence, it may cover only some parts of a gene, leaving other parts without evidence. Traditional *ab initio* gene prediction methods rely on computational predictions by statistical models using genome sequence data alone, for example AUGUSTUS [Stanke et al., 2006] and GeneMark-ES [Lomsadze et al., 2005]. However, the *ab initio* models are prone to errors when used alone. Therefore, a higher accuracy of gene predictions is achieved when predictions based on statistical models are corrected by extrinsic evidence [Stanke et al., 2008, Lomsadze et al., 2014, Brůna et al., 2020].

Earlier developed BRAKER1 [Hoff et al., 2016] and BRAKER2 [Brůna et al., 2021] combined GeneMark and AUGUSTUS to utilize, respectively, a single source of extrinsic evidence, either RNA-Seq short reads or homologous proteins. The use of both extrinsic evidence sources together has a clear potential for more accurate gene structure prediction. Therefore, we developed a combiner tool TSEBRA [Gabriel et al., 2021]. It selects transcripts from BRAKER1 and BRAKER2 annotations, considering thereby the joint extrinsic evidence and, therefore, generates a prediction based on both RNA-Seq and protein evidence, thus improving the gene prediction accuracy.

A more integrated approach is the GeneMark-ETP pipeline [Brůna et al., 2023], which integrates both sources of extrinsic evidence in a new workflow that outperforms all previously mentioned methods, particularly in species with large and complex genomes. Critical to its improvement are a novel approach to generate a highly specific training set from genes predicted in assembled transcripts and supported by protein evidence. The method also benefited from the GC-content-specific model training, and estimating species-specific repeat penalties.

These many advancements and the popularity of the previous BRAKER tools motivated us to develop a new version of the BRAKER pipeline that can utilize both transcript and protein homology extrinsic evidence by incorporating GeneMark-ETP, AUGUSTUS, and TSEBRA into a novel workflow.

Similar tools that use RNA-Seq and protein data are MAKER2 [Holt and Yandell, 2011], FINDER [Banerjee et al., 2021] and Funannotate (GitHub, Palmer [2017]). MAKER2 aligns assembled RNA-Seq data and proteins to the genome and can run and integrate SNAP [Korf, 2004], GeneMark and AUGUSTUS predictions. Although MAKER2 can provide training sets for SNAP and AUGUSTUS, it does not train the *ab initio* models automatically. Also, the self-training of GeneMark.hmm models [Lomsadze et al., 2005] has to be done outside of MAKER2. FINDER follows an approach similar to BRAKER3. It uses RNA-Seq assemblies with predicted open reading frames, in conjunction with BRAKER1 and homologous protein predictions. The Funannotate pipeline, which was not described in an article, was initially designed as a pipeline for analyzing fungal genomes; however, it has since been further developed to support the annotation of larger genomes as well [Palmer, 2017].

## Methods

### BRAKER3

BRAKER3 is the latest pipeline in the BRAKER suite. It requires three types of inputs: the genome sequence to annotate, a list of short read RNA-Seq datasets and a protein database file. The protein database is a FASTA file with proteins from the broad clade of the target genome in question, e.g., a subset from the partitioning of OrthoDB that we provide (see Suppl. Methods). To specify the RNA-Seq input there are three options, as BAM-files of aligned reads, raw reads in FASTQ-files, or SRA (Leinonen et al. [2010]) library IDs.

The data for generating external hints is processed by GeneMark-ETP. Similar to GeneMark-EP+, intron hints, as well as start and stop codon hints, are created by ProtHint from spliced alignments of database proteins to the genome [Brůna et al., 2021]. Similarly to GeneMark-ET, intron hints are created by spliced alignments of RNA-Seq to genome by HISAT2 [Kim et al., 2019]. Notably, a new type of external hints is created from assembled StringTie2 transcripts [Kovaka et al., 2019]. Protein-coding genes are predicted in assembled transcripts by GeneMarkS-T [Tang et al., 2015]. The level of confidence in such a prediction is determined on the basis of the alignment of the predicted protein to the proteins in the reference database. Out of these predictions we select those that have high similarity scores. Besides these types of gene predictions, some other genes predicted in transcripts are selected based on the quality of *ab initio* predictions and other criteria as described in the description of GeneMark-ETP [Brůna et al., 2023]. This set of high-quality gene predictions in assembled transcripts gives rise to a set of High Confidence (HC) genes. Overall, GeneMark-ETP creates three distinct groups of the external hints: external hints with both transcript and protein similarity support, hints with transcript and *ab initio* support and hints supported by protein similarity only (generated by ProtHint). All these sets are used for training of the statistical model and expanding of the set of HC genes to a set of genome wide gene prediction by GeneMark-ETP.

**Figure 1:**
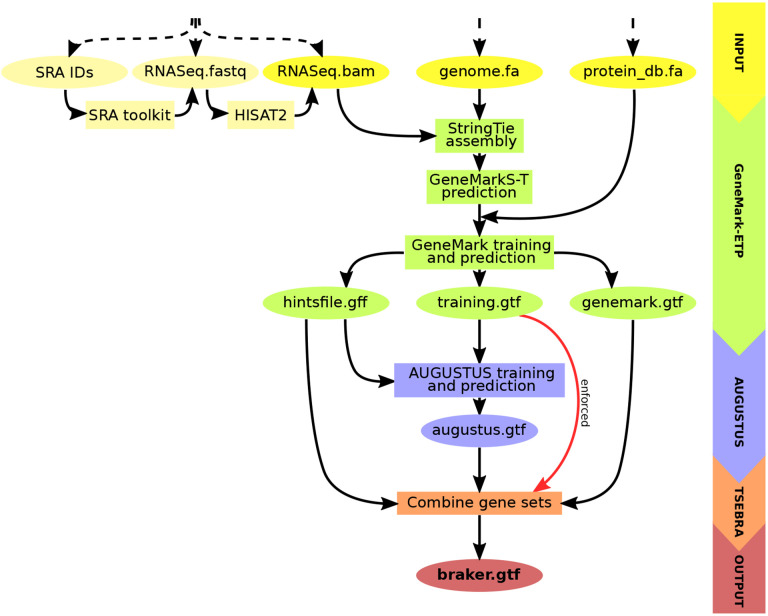
Schematic view of the BRAKER3 pipeline. Required inputs are genomic sequences, short-read RNA-Seq data, and a protein database. The RNA-Seq data can be provided in three different forms: IDs of libraries available at the Sequence Read Archive [Leinonen et al., 2010], unaligned reads or aligned reads. If library IDs are given, BRAKER3 downloads the raw RNA-Seq reads using the SRA Toolkit [SRA Toolkit Development Team, 2020] and aligns them to the genome using HISAT2 [Kim et al., 2019]. It is also possible to use a combination of these formats when using more than one library.

At the next step, AUGUSTUS is trained on a set of HC genes and predicts a second genome wide gene set with the support of external hints. At the final step, an updated TSEBRA (described next) combines the predictions made by AUGUSTUS and GeneMark-ETP while integrating the HC genes directly into the result to ensure their inclusion. The workflow is illustrated in Figure 1.

### TSEBRA

The Transcript SElector for BRAker (TSEBRA) was improved and its original use in the BRAKER suite was extended. As described earlier, TSEBRA combines gene sets by evaluating and comparing candidate transcript isoforms using four transcript scores, which measured the agreement of transcript structures with extrinsic evidence. The extrinsic evidence is here utilized in the form of positions of supported exon borders, particularly intron position intervals. We have now introduced normalization of these transcript scores with respect to all input gene sets to TSEBRA, so the support with evidence is measured relative to the available evidence for the target genome. Normalization of a transcript score *s* for the *i*-th transcript of the input gene sets is defined as: $s_{\text{norm}}^{i}:=\left(s^{i}-\mu_{s}\right)/\sigma_{s}$, where *μ*_*s*_ and *σ*_*s*_ are the average and standard deviation of one of four transcript score measures *s*, calculated from scores of all transcripts in the input gene sets that TSEBRA is requested to combine. TSEBRA heavily relies on intron position information, which can make it challenging to evaluate single-exon transcripts. Therefore, the original TSEBRA tended to overestimate single-exon transcripts in some cases. To address this, we added a new option to TSEBRA that allows filtering out those single-exon genes that are predicted without any support by start- or stop-codon hints. When run by BRAKER3 on genomic sequences longer than 300Mbp, TSEBRA removes such single-exon genes that are predicted purely *ab initio*.

We also added TSEBRA to the workflow of BRAKER1 and BRAKER2, where it is now used to combine AUGUSTUS predictions with transcripts from GeneMark-ET/EP that are highly supported by extrinsic evidence.

### Test data

We compiled test data for accuracy assessment experiments for 11 target species. For each species, we retrieved: genome assemblies, 5 or 6 randomly selected short-read RNA-Seq libraries (detailed list in Suppl. Table 13), a protein database, and a reference genome annotation (detailed list in Suppl. Table 1). Before running the experiments, we soft-masked repeats in the genomic sequences using RepeatModeler2 [Flynn et al., 2020].

For each target species, we prepared three protein databases containing sequences of the same taxonomic clade. The first two databases are large and include a large and broad partition of OrthoDB (*Arthropoda*, *Metazoa*, *Vertebrata* or *Viridiplantae* depending on the target species). With them we benchmark the scenario in which most possibly useful available proteins are used as informants. To test the influence of the proximity of the most closely related informant species to the target species, we compiled two database variants for each target species. In one series of databases, only the protein sequences from the target species itself were removed (*species excluded*), and in the other the proteins of all species of the same taxonomic order were removed (*order excluded*).

Funannotate failed to run on most of these large protein databases. It runs the protein spliced aligner Exonerate [Slater and Birney, 2005] in a way that appears to be problematic for large database inputs. On a cluster with 189 GB RAM per node and 72h job limit Funannotate ran successfully only for two species. MAKER2 was designed to be used with a smaller protein database, too. Therefore, to allow three-way comparisons of BRAKER3 with MAKER2 and Funannotate on the same input data we compiled a third series of informant protein databases we call *close relatives included*: For each species a small number of 4–12 closely related species was selected and all their proteins included. These databases are smaller than the corresponding *species excluded* and *order excluded* databases by a factor between 17 and 132. It should be noted that the species for the *close relatives included* database were manually selected and the procedure would not scale well when very large numbers of species are annotated. Suppl. Table 14 lists the species that were included in the *close relatives included* databases.

### Experiments

We evaluated the performance of BRAKER3 and compared it with seven other methods: the previous versions BRAKER1 and BRAKER2 using only one type of extrinsic evidence (as included in the BRAKER v3.0.2 suite), TSEBRA (v1.1.0), combining the results of BRAKER1 and BRAKER2, MAKER2 (3.01.04), FINDER (v1.1.0), Funannotate (v1.8.14), and GeneMark-ETP. As BRAKER3, BRAKER2, FINDER and GeneMark-ETP can use a large protein database and since doing so saves a manual step we compared these four tools along with BRAKER1 and TSEBRA in two sets of experiments, where the large *order excluded* and *species excluded* databases were used. In another set of experiments, we compared BRAKER3 with MAKER2 and Funannotate on the smaller and target-specific *close relatives included* databases using the same RNA-Seq data as in the other experiments.

When running Funannotate, we tried two recommended flags for generating gene sets, a specific handling of repetitive regions and an additional gene model update step. This resulted in four variant sets of gene predictions per genome. Here, we report the numbers of the variant of Funannotate that performed best (both flags were set, Suppl. Table 7).

MAKER2 was executed according to recommendations provided by the developers of MAKER2, integrating GeneMark, AUGUSTUS and SNAP predictions. The details are provided in the Supplementary Material. MAKER2 does not provide automatic training procedures. A recommended approach is the manual execution of training runs of all the *ab initio* programs outside of MAKER2. To provide the best possible models, we trained SNAP and AUGUSTUS on the respective reference annotation which all programs were evaluated on, unless models for SNAP or AUGUSTUS for the species were included in the standard distribution of these tools. Models for GeneMark were also chosen to match the best possible training routine (see Supplementary Material). This approach allowed for automatic execution of MAKER2. However, the quality of the trained parameters of the gene finders we used for MAKER2 can be considered as rather as *upper limits of what can be expected* on new genomes.

We compared the predicted genome annotations with the reference annotations to assess the accuracy of BRAKER3 on exon, gene and transcript levels. As metrics for accuracy, we used the sensitivity (Sn) - the percentage of correctly found instances from the reference annotation, the specificity (Sp) - the percentage of correct instances in the predicted annotation, and the F1-score - the harmonic mean of Sn and Sp. When evaluating exon level or transcript level accuracy, each transcript / exon was individually assessed. However, when evaluating gene level accuracy, a predicted gene was counted as true positive if at least one of its predicted alternative transcripts matched a reference transcript.

## Results and discussion

### Using large protein databases

**Figure 2:**
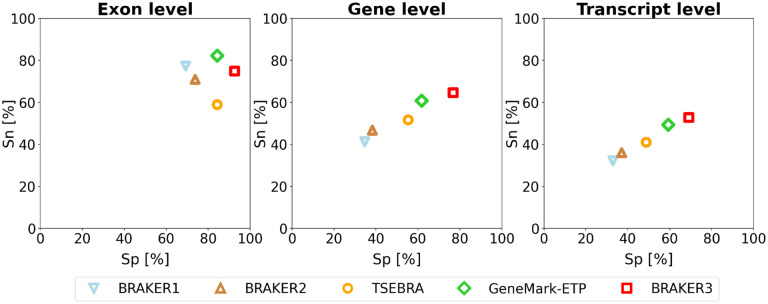
Average specificity and sensitivity of gene predictions made by BRAKER1, BRAKER2, TSEBRA, GeneMark-ETP, and BRAKER3 for the genomes of 11 different species (listed in Suppl. Table 1). Inputs were the genomic sequences, short-read RNA-Seq libraries, and protein databases (*order excluded*).

The *order excluded* protein database yields a more conservative accuracy estimate than the *species excluded* set and is arguably a more realistic scenario for novel eukaryotic genomes for which no close species with a trusted annotation are yet available. Figure 2 shows the accuracies averaged over the 11 genomes. The pipelines in order of increased accuracy are BRAKER1, BRAKER2, TSEBRA combining BRAKER1+2, GeneMark-ETP and BRAKER3. Suppl. Table 3 shows the accuracy values for individual genomes, as well as the averaged values for the 11 genomes.

**Figure 3:**
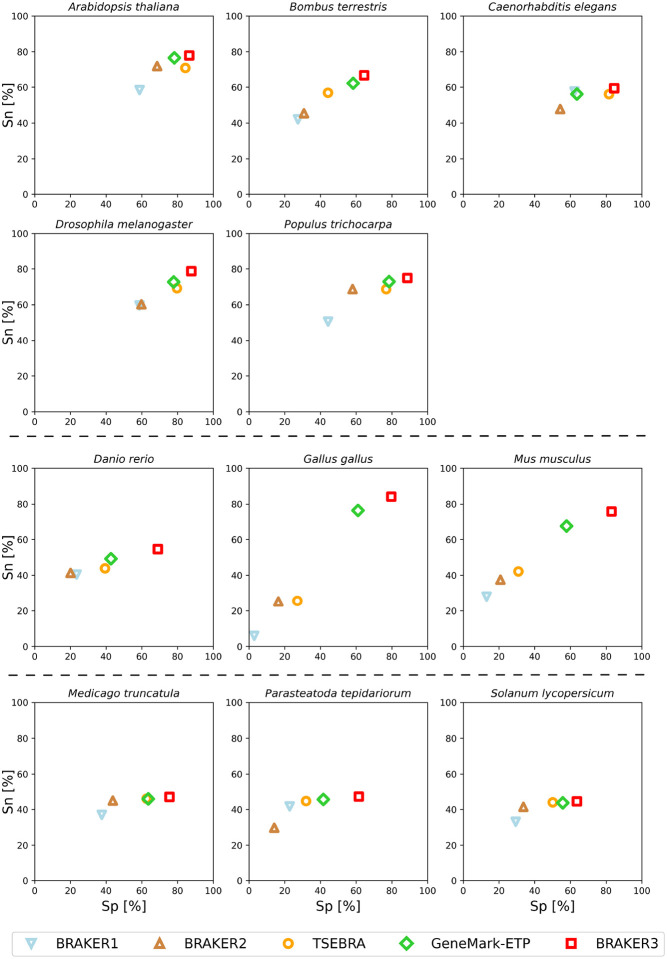
Gene level specificity and sensitivity of gene predictions made by BRAKER1, BRAKER2, TSEBRA, GeneMark-ETP, and BRAKER3 for the genomes of 11 different species: well annotated and compact genomes (first and second row), well-annotated and large genomes (third row), other genomes (fourth row). Inputs were the genomic sequences, short-read RNA-Seq libraries, and protein databases (*order excluded*).

Notably, there was a significant improvement in accuracy of BRAKER3 in comparison with BRAKER1 and BRAKER2 in species with GC-heterogeneous or large genomes (Figure 3). The highest increase in accuracy was achieved in *Gallus gallus*, where the BRAKER3 F1-score on gene / transcript level was improved by 55/48 points compared to the combined prediction of BRAKER1 and BRAKER2 generated by TSEBRA (Suppl. Table 2). Here, BRAKER3 greatly benefited from the improved accuracy of GeneMark-ETP and managed to exceed the accuracy even further. GeneMark-ETP enabled the generation of a highly specific set of training genes (HC genes) to train the AUGUSTUS model. As a result, this AUGUSTUS prediction using extrinsic evidence of BRAKER3 had an even higher sensitivity than GeneMark-ETP and average gene and transcript level F1-scores of 59.6 and 51.3, respectively, which exceeded the accuracies of the AUGUSTUS predictions in BRAKER1 and BRAKER2, and is comparable to the accuracy of GeneMark-ETP, see Suppl. Table 12. By integrating TSEBRA into BRAKER3 and combining sets of gene predictions made by GeneMark-ETP and AUGUSTUS, the final BRAKER3 predictions achieved higher sensitivity and specificity at both the gene and transcript level.

When we used the *species excluded* protein database, which may include very closely related species, the accuracy of the methods using the protein data increased overall (Suppl. Table 4). On average, the BRAKER3 transcript level sensitivity was improved by approximately 3 percentage points and the specificity was improved slightly (less than 1 percentage point). However, the relative ranking of the methods and the comparison of BRAKER3 with other methods remain unchanged.

The FINDER annotation pipeline was run on the *order excluded* databases with the same input data as BRAKER3, but the execution only completed for 7 out of 11 species. Still, its best performance, a ~15/11 gene / transcript F1-score for *Drosophila melanogaster*, was much below the accuracy of the other methods (Suppl. Table 11). Possible reasons for the observation that the performance of FINDER was below the figures published by Banerjee et al. [2021] are that these authors did not exclude any proteins from their UniProt informant database (Sagnik Banerjee, personal communication) and that Banerjee et al. used many more RNA-Seq libraries.

BRAKER3 had the highest accuracy for each species at the transcript and gene level, but often had a somewhat lower exon-level F1-score than GeneMark-ETP (Suppl. Table 3). In each species, BRAKER3 was more specific in predicting exons than GeneMark-ETP, which in turn predicted exons more sensitively than BRAKER3 (Suppl. Table 2). Thus, there was a trade-off in exon sensitivity and specificity between the two methods, with an average difference of approximately 8 percentage points in both measures (Suppl. Table 2). We presume that the occasional false-positive exons of GeneMark-ETP hurt the stricter transcript and gene accuracy measures more than those exons occasionally missed by BRAKER3 do.

### Using small protein databases

In the *close relatives included* setting, Funannotate and MAKER2 did not complete successfully the annotation of all 11 genomes. The runs of Funannotate for *Mus musculus* and *Parasteatoda tepidariorum* resulted in out-of-memory errors, even for the smaller protein sets. MAKER2 did not finish on the zebrafish genome. We therefore compared the outcomes only for the eight species where both MAKER2 and Funannotate succeeded in running.

**Figure 4:**
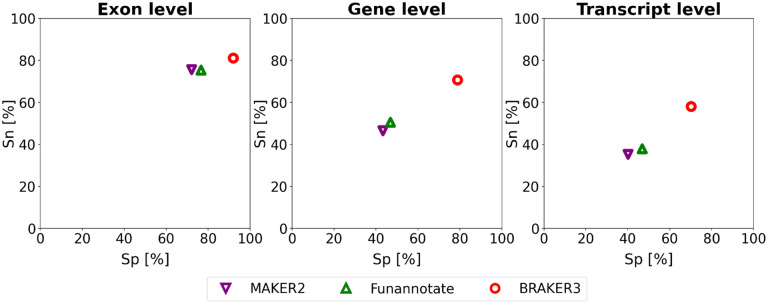
Average specificity and sensitivity of gene predictions made by MAKER2, Funannotate, and BRAKER3 for a subset of 8 species (excluding the mouse, spider an fish genome). Inputs were the genomic sequences, short-read RNA-Seq libraries, and protein databases (*close relatives included*). MAKER2 was here given an advantage to avoid manual training steps, but this option is not available on new genomes. Therefore this accuracy of MAKER2 can be regarded as an upper limit of what can be expected when annotating a previously unannotated genome (see Experiments section).

Figure 4 and Suppl. Table 6 show the comparison of BRAKER3 to MAKER2 and Funannotate. All tools, including BRAKER3, are given as input the smaller *close relatives included* protein databases and the same RNA-Seq data as in all experiments. BRAKER3 consistently exceeds the accuracy of Funannotate and MAKER2 on exon, gene and transcript level (Figure 4). On average, BRAKER3’s F1-score was 10.2 points at the exon level, 25.9 points at the gene level and 21.6 points at the transcript level higher than Funannotate’s F1-score. In turn Funannotate exceeded MAKER2 by 2.2, 3.8 and 4.4 points with regards to the F1 measure on exon, gene and transcript level, respectively. BRAKER3 exceeds the accuracy of Funannotate and MAKER2 for all species and individual metrics, except at the exon level for *Caenorhabditis elegans*, where BRAKER3 had a lower sensitivity by 3.3 percentage points (Suppl. Table 6).

As the accuracy of a pipeline does not necessarily have to improve when more external evidence is used, which may contain more remote proteins, we compared the results of BRAKER3 on the protein informant databases *close relatives included* and *species excluded*. Both series of databases may contain proteins from close relatives of the target, but the database *close relatives included* is much smaller. When run with the *species excluded* database BRAKER3 has on average an F1-score that is higher by 0.43, 0.04 and 0.16 on the exon, gene and transcript level, respectively, than when BRAKER3 is run with the *close relatives included* database. Thus, when BRAKER3 uses the larger protein database it delivers slightly better results and has a practical advantage in that it does not require the (manual) step to compile a database of closely related proteomes.

### Runtime

We ran all methods but MAKER2 on an HPC node with Intel(R) Xeon(R) CPU E5–2650 v4 @ 2.20GHz using 48 threads. The pipelines in order of runtime when using the *order excluded* databases were BRAKER1, GeneMark-ETP, BRAKER2, BRAKER3. The BRAKER3 runtime ranges from 5h 37min in *Arabidopsis thaliana* to 64h 16min in *Mus musculus*. Despite having the longest run-time of all methods, BRAKER3 can annotate even large genomes in a reasonable time. BRAKER3 required only 23% more time on the large protein databases compared to the much smaller *close relatives included* protein databases (averaged over 8 species, compare Suppl. Tables 8 and 10).

We also compared the runtimes of BRAKER3, Funannotate and MAKER2 on the smaller *close relatives included* protein databases. Funannotate required on average roughly the same time as BRAKER3 (see Supp. Table 10). As we ran MAKER2 on faster hardware (see Supplementary Methods) we made a runtime comparison experiment with BRAKER3 on the same hardware for *Drosophila melanogaster*. When given the relatively small protein database as input (116,493 proteins) MAKER2 took 2.1 hours and BRAKER3 took 3.5 hours. When given a large protein database as input (2,588,444 proteins), MAKER2 took 16 hours and BRAKER3 took 2.5 hours. The run-time of BRAKER3 is much less dependent on the protein database size (here its decrease was due to a variable-duration hyperparameter optimization step during training). When comparing these runtimes, one has to consider that the figures for MAKER2 do not include the considerable times for training gene finders and neither for transcript-tome assembly. In contrast, BRAKER3 performs these steps as part of the pipeline. Some further examples of runtimes of MAKER2 are shown in Supp. Table 9.

### Virtualization

One problem with modern genome annotation pipelines is their dependence on an increasing number of tools, which can make their installation and maintenance difficult. Therefore, we provide a Singularity [Kurtzer et al., 2017] container for BRAKER, making it easy to install and use.

## Conclusion

We present BRAKER3, a novel genome annotation pipeline for eukaryotic genomes that integrates evidence from transcript reads, homologous proteins and the genome itself. We report significantly improved accuracy for 11 test species. BRAKER3 outperforms its predecessors BRAKER1 and BRAKER2 by a large margin, as well as publicly available pipelines, such as MAKER2, FINDER and Funannotate. The most substantial improvements are observed in species with large and complex genomes. Additionally, BRAKER3 adds a Singularity container to the BRAKER suite, which makes it more user-friendly and easier to install.

## Supplementary Material

Supplement 1

## Data Availability

BRAKER3 is available on GitHub (https://github.com/Gaius-Augustus/BRAKER) and as a Docker/Singularity container (https://hub.docker.com/r/teambraker/braker3). All data was previously publicly available. Genome versions, repeat masking and annotation processing are documented at https://github.com/gatech-genemark/EukSpecies-BRAKER2 and at https://github.com/gatech-genemark/GeneMark-ETP-exp.
