# Supplementary material for: BRAKER3: Fully automated genome annotation using RNA-seq and protein evidence with GeneMark-ETP, AUGUSTUS and TSEBRA
Source: bioRxiv. 2024 Feb 29:2023.06.10.544449. Originally published 2023 Jun 12. Preprint. [Version 4] doi: 10.1101/2023.06.10.544449 (PMC10312602; doi:10.1101/2023.06.10.544449)
Supplement: Supplement 1 [file media-1.pdf]

1 Supplemental Material

1.1 Supplemental Figures

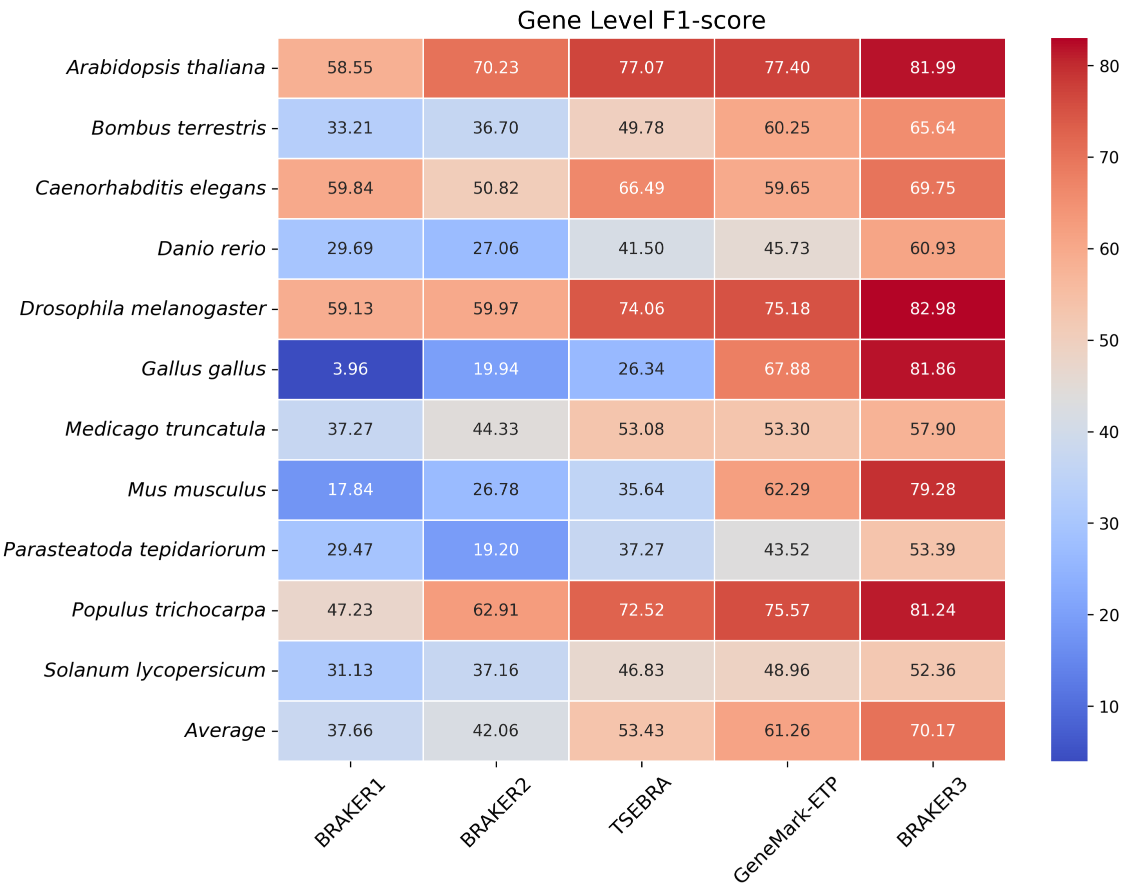

Supplemental Figure S1: Heatmap of F1-scores of pipelines being input short-read RNA-seq libraries and a protein database (with proteins of species from the respective **order excluded**). The last row shows the averages for the 11 different species.

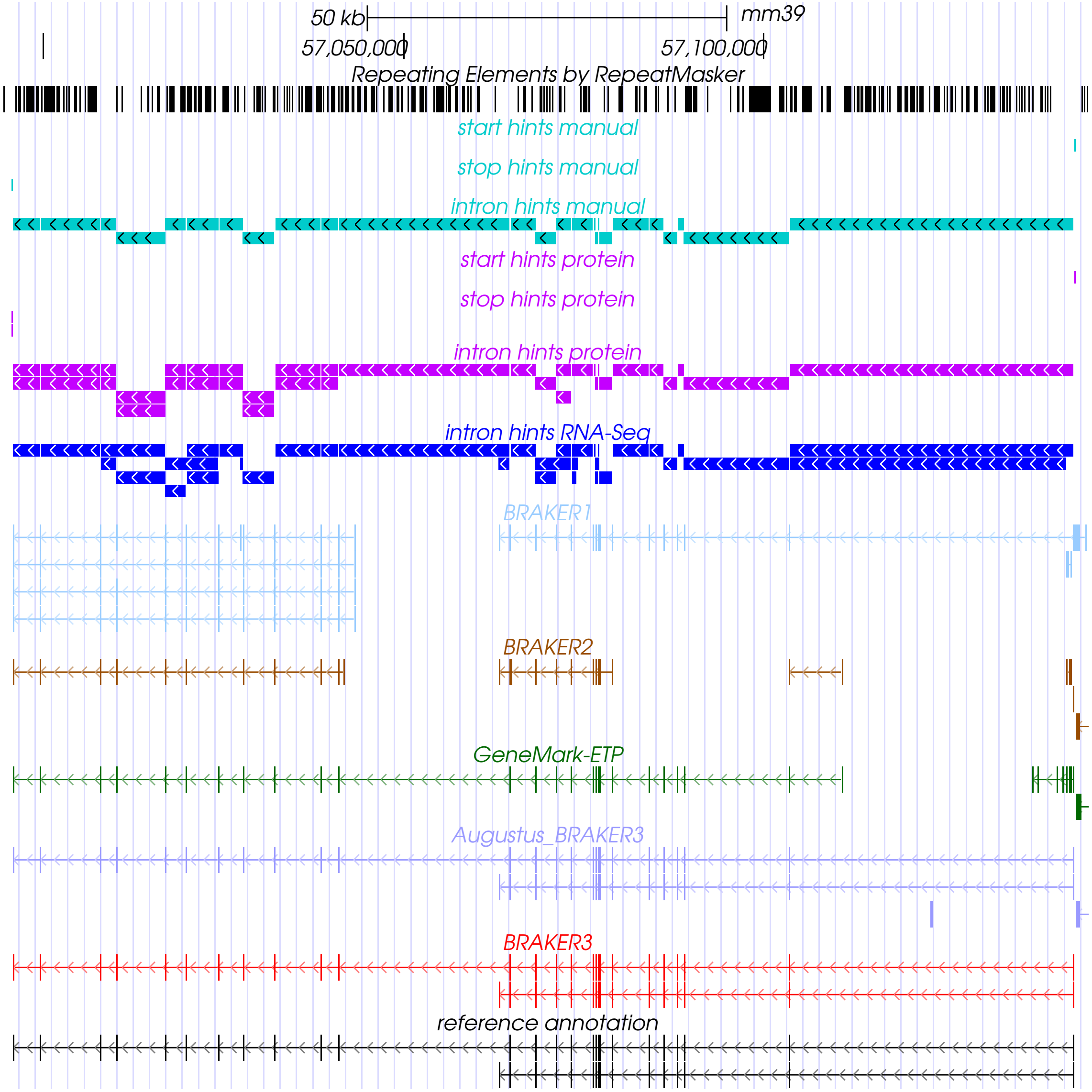

Supplemental Figure S2: A gene locus of *Mus musculus* visualized with the UCSC Genome Browser (Kent et al. (2002), `http://genome.ucsc.edu`). Tracks for the gene prediction of pipelines obtaining short-read RNA-seq libraries and a protein database (respective *order excluded*) as input are displayed. Tracks highlighting exon boundaries based on extrinsic evidence are shown at the top. Manual hints represent high confidence hints that are highly weighted during AUGUSTUS prediction. In this example, TSEBRA selects two correct transcripts from AUGUSTUS run as part of BRAKER3 (AUGUSTUS_BRAKER3) and filters out three false transcripts from GeneMark-ETP and two from AUGUSTUS.

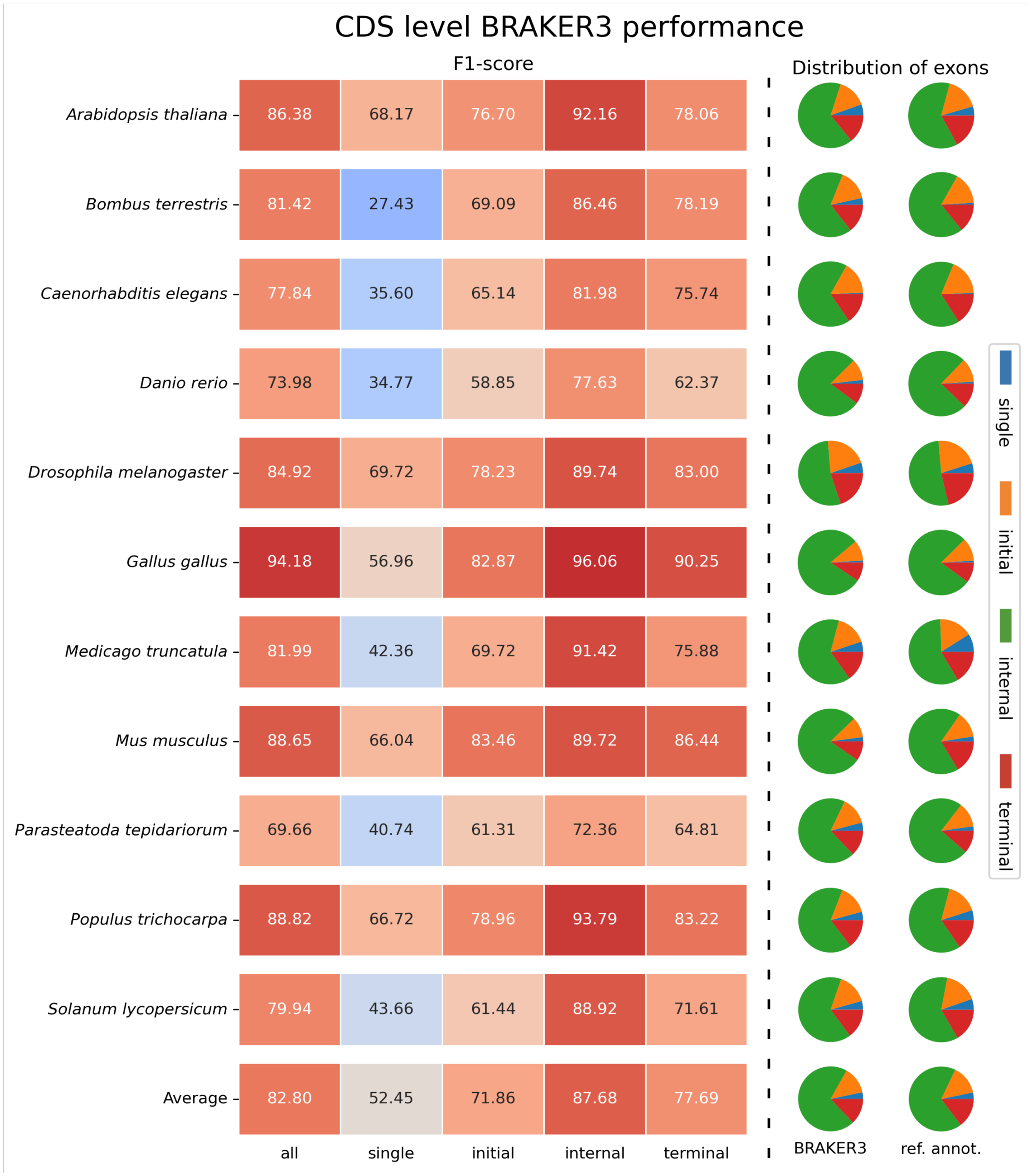

Supplemental Figure S3: The F1 score when predicting the protein-coding (parts of) exons, broken down by type of exon. Single: unspliced coding region, initial/terminal: first/last coding region, respectively, of a gene with spliced coding sequence, internal: coding region bordered by a splice site on both sides. The pie charts visualize the proportions of exon types in the BRAKER3 and reference annotations.

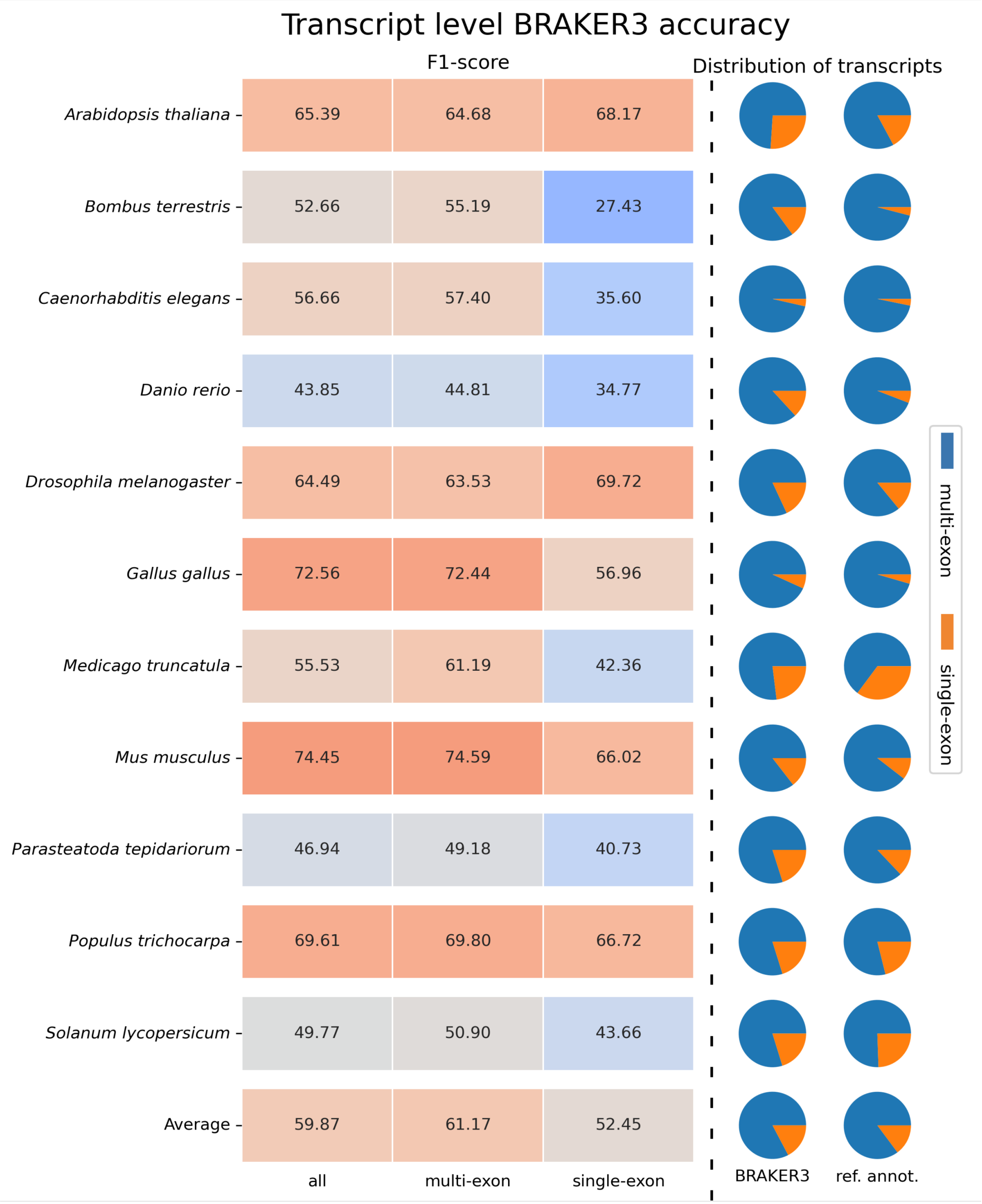

Supplemental Figure S4: Performance of BRAKER3 broken down by whether the coding sequence of a transcript is spliced (multi-exon) or unspliced (single-exon).

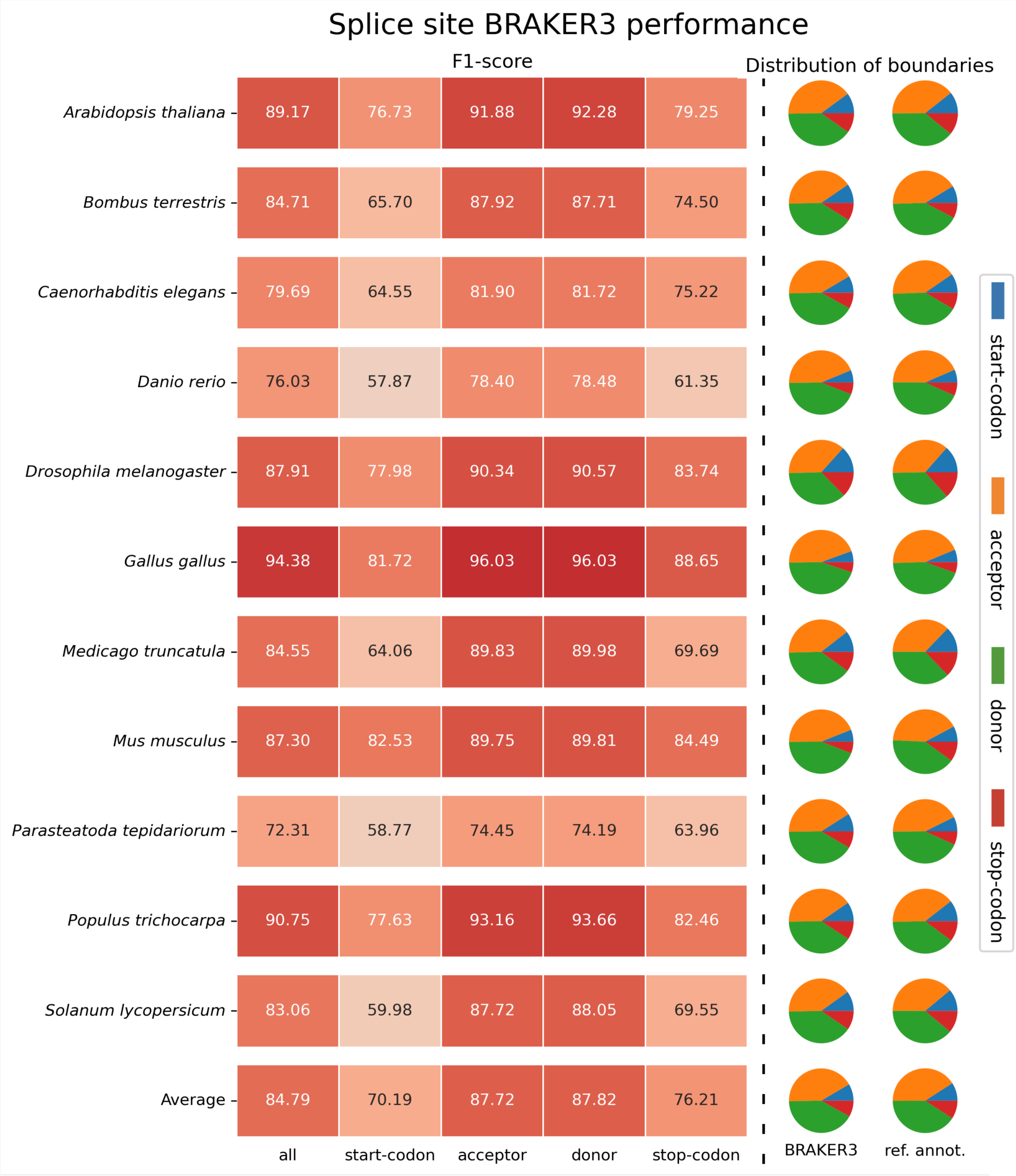

Supplemental Figure S5: F1-score for predicting the signal at the boundaries of coding regions, start- and stop codons and acceptor/donor splice sites, downstream/upstream of an intron, respectively.

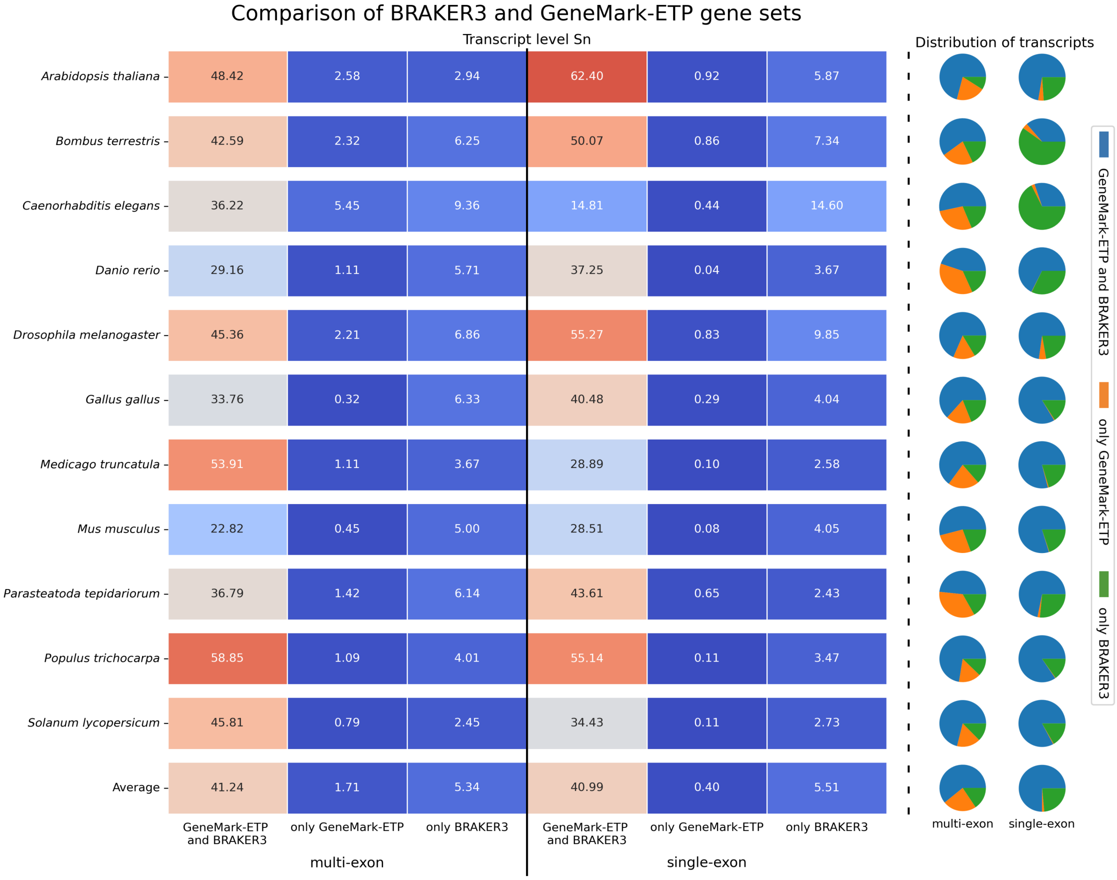

Supplemental Figure S6: The left side of the heat map refers to spliced genes, the right side to unspliced genes. The cells show the percentage of reference transcripts that are correctly predicted by both BRAKER3 and GeneMark-ETP (left columns), by GeneMark-ETP only (middle columns) and by BRAKER3 only (right columns). The pie charts to the right visualize the relative sizes of the predicted transcript sets regardless of whether they are correct.

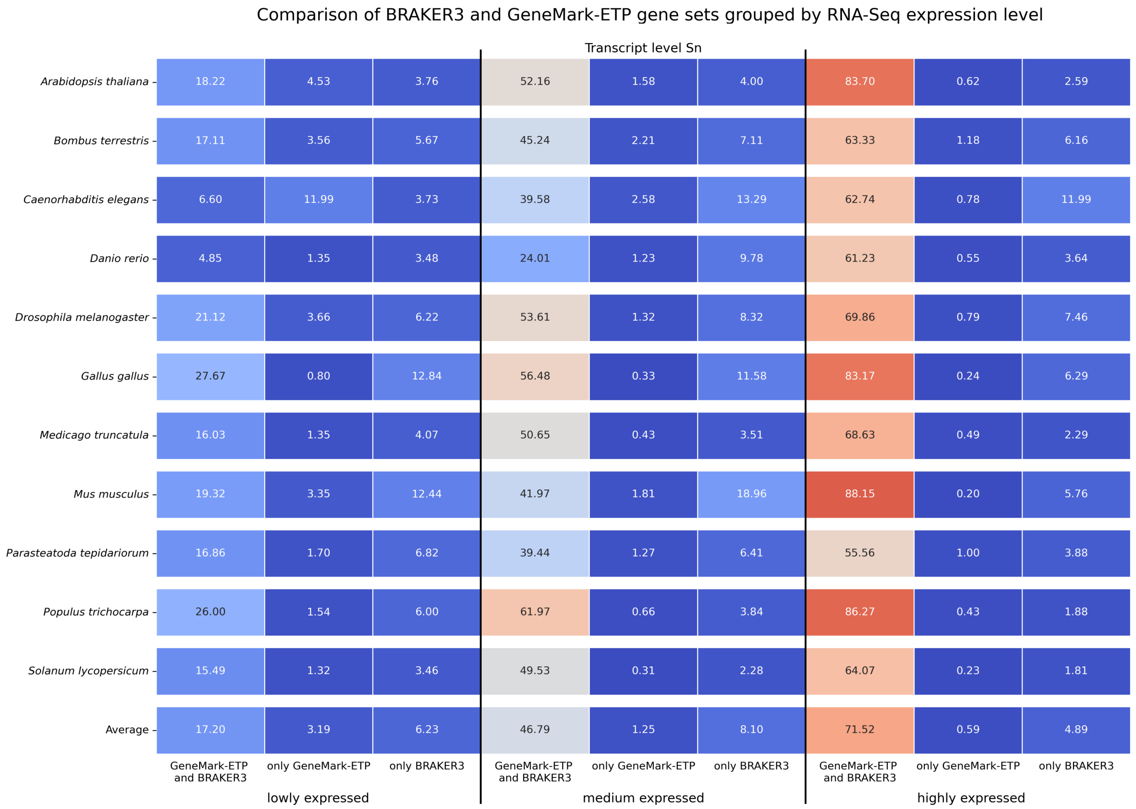

Supplemental Figure S7: The heat map shows the percentage of reference transcripts, in the first, second and third expression tercile, that are correctly identified by BRAKER3 and GeneMark-ETP or by either program only.

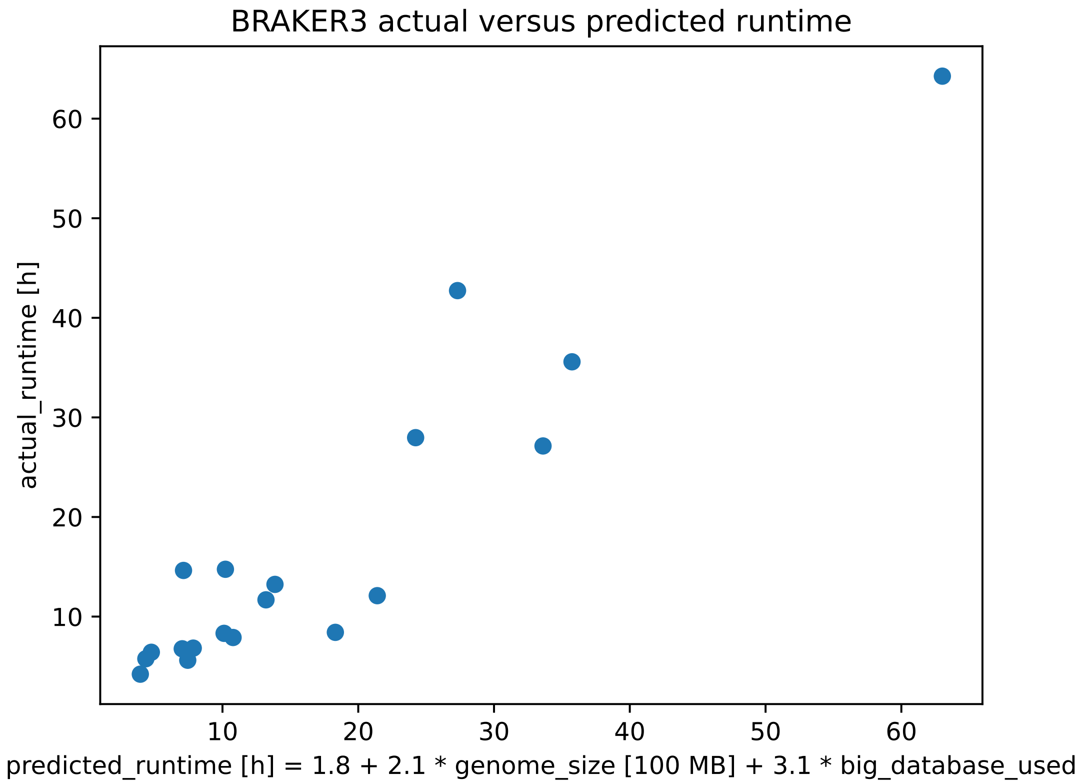

Supplemental Figure S8: Actual runtime versus the runtime predicted for 19 whole genomes. The regression to predict the runtime ($R^2 = 0.87$) considered only the size of the genome and whether an OrthoDB partition was used (`big_database_used`=1) or only the proteomes of a few closely related genomes were used (`big_database_used`=0).

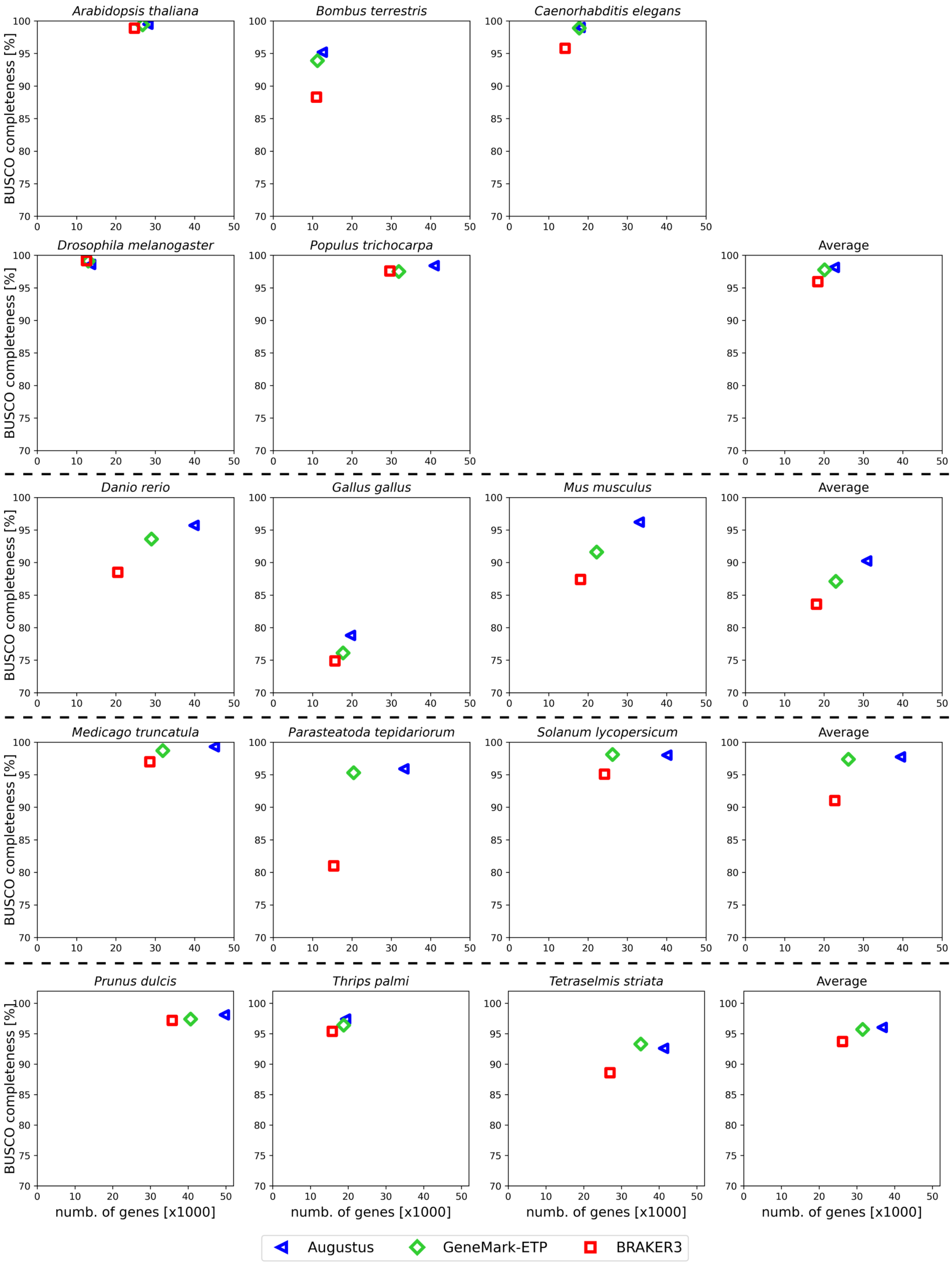

Supplemental Figure S9: Proteome completeness versus the number of genes. The horizontal axis shows the total gene count, after alternative transcripts were grouped into genes. The vertical axis shows the BUSCO completeness percentage (single-copy or duplicated) for the respective gene sets. The AUGUSTUS and GeneMark-ETP gene sets were taken from the output of BRAKER3.

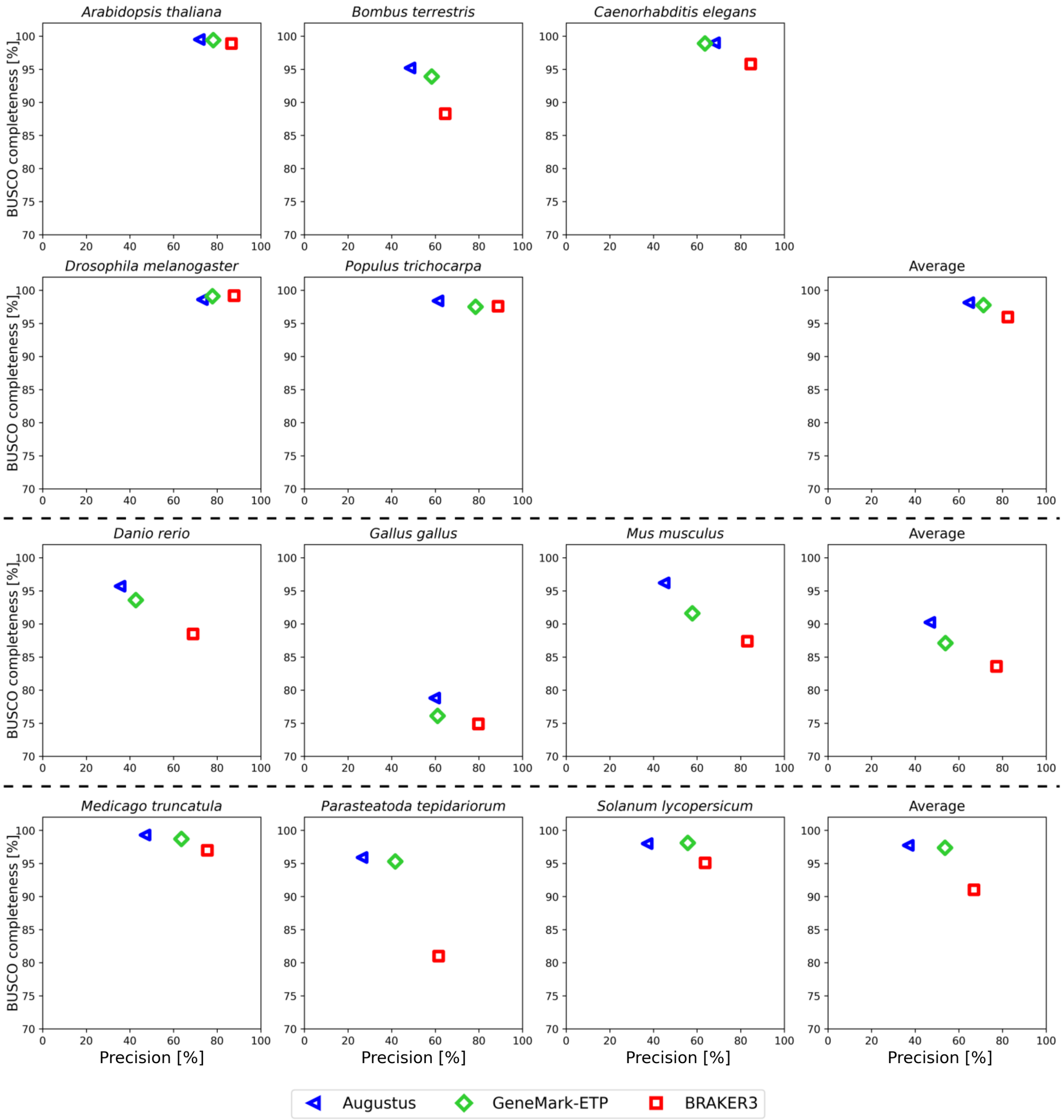

Supplemental Figure S10: Proteome completeness versus gene-level precision. The data is as in Supplemental Figure S9, except that the horizontal axis shows the percentage of predicted genes that identically share a transcript with the reference annotation.

1.2 Supplemental Tables

Species	Annotation version	Size (Mbp)	#Genes	#Transcripts	#CDS	# Sequences in protein database		
						species excluded	*order excluded*	*close relatives included*
Arabidopsis thaliana	TAIR Araport 11 (Jun 2016)	119	27,445	48,149	156,731	5,283,100	4,825,128	306,719
Bombus terrestris	NCBI Annotation Release 102 (Apr 2017)	249	10,581	22,091	78,337	4,297,173	3,416,393	180,811
Caenorhabditis elegans	WormBase WS271 (May 2019)	100	20,172	33,624	130,885	15,237,008	15,145,380	115,553
Danio rerio	Ensembl GRCz11.96 (May 2019)	1,345	25,611	42,934	262,325	9,779,764	9,468,332	760,754
Drosophila melanogaster	FlyBase R6.18 (Jun 2019)	138	13,930	30,561	62,841	4,293,925	3,029,616	116,493
Gallus gallus	Ensembl GRCg6a.105 (March 2018)	1,050	17,279	38,534	192,095	9,787,814	9,690,812	220,641
Medicago truncatula	MtrunA17r5.0-ANR-EGN-r1.6 (Feb 2019)	420	44,464	44,464	174,281	5,278,627	4,680,430	134,774
Mus musculus	GENCODE GRCm39 version M28	2,723	22,405	58,318	243,366	9,782,804	9,059,968	510,476
Parasteatoda tepidariorum	NCBI Annotation Release 101 (May 2017)	1,445	18,602	27,516	143,792	4,287,893	4,240,214	1,163,197
Populus trichocarpa	JGI Ptrichocarpa_533_v4.1 (Nov 2019)	389	34,488	52,085	187,170	5,278,879	5,112,927	199,662
Solanum lycopersicum	Consortium ITAG4.0 (May 2019)	773	33,562	33,562	152,352	5,284,979	4,823,899	149,444

Supplemental Table S1: Summary of genomes, annotations and protein databases used for performance evaluation. Data extracted from Table 4 in Brůna et al. (2023b) and computed from raw data of Brůna et al. (2021, 2023b). Genome versions, repeat masking and annotation processing are documented at `https://github.com/gatech-genemark/EukSpecies-BRAKER2` and at `https://github.com/gatech-genemark/GeneMark-ETP-exp`. The protein databases were generated with orthodb-clades from `https://github.com/tomasbruna/orthodb-clades`.

**Species**	**Reference Protein File**
Arabidopsis thaliana	
Arabidopsis lyrata subsp. lyrata	GCF_000004255.2_v.1.0_protein.faa.gz
Arabidopsis thaliana x Arabidopsis arenosa	GCA_019202795.1_ASM1920279v1_protein.faa.gz
Camelina sativa	GCF_000633955.1_Cs_protein.faa.gz
Arabidopsis suecica	GCA_019202805.1_ASM1920280v1_protein.faa.gz
Capsella rubella	GCF_000375325.1_Caprub1_0_protein.faa.gz
Bombus terrestris	
Bombus vancouverensis nearcticus	GCF_011952275.1_Bvanc_JDL1245_protein.faa.gz
Bombus huntii	GCF_024542735.1_iyBomHunt1.1_protein.faa.gz
Bombus affinis	GCF_024516045.1_iyBomAffi1.2_protein.faa.gz
Bombus pyrosoma	GCF_014825855.1_ASM1482585v1_protein.faa.gz
Bombus vosnesenskii	GCF_011952255.1_Bvos_JDL3184-5_v1.1_protein.faa.gz
Bombus bifarius	GCF_011952205.1_Bbif_JDL3187_protein.faa.gz
Bombus impatiens	GCF_000188095.3_BIMP_2.2_protein.faa.gz
Caenorhabditis elegans	
Caenorhabditis auriculariae	GCA_904845305.1_CAUJ_protein.faa.gz
Caenorhabditis bovis	GCA_902829315.1_CBOVIS_v1.1_protein.faa.gz
Caenorhabditis brenneri	GCA_000143925.2_C_brenneri-6.0.1b_protein.faa.gz
Caenorhabditis briggsae	GCF_000004555.2_CB4_protein.faa.gz
Caenorhabditis remanei	GCF_000149515.1_ASM14951v1_protein.faa.gz
Danio rerio	
Cyprinus carpio	GCF_018340385.1_ASM1834038v1_protein.faa.gz
Carassius auratus	GCF_003368295.1_ASM336829v1_protein.faa.gz
Puntigrus tetrazona	GCF_018831695.1_ASM1883169v1_protein.faa.gz
Sinocyclocheilus rhinocerous	GCF_001515625.1_SAMN03320098_v1.1_protein.faa.gz
Sinocyclocheilus anshuiensis	GCF_001515605.1_SAMN03320099.WGS_v1.1_protein.faa.gz
Onychostoma macrolepis	GCA_012432095.1_ASM1243209v1_protein.faa.gz
Carassius gibelio	GCF_023724105.1_carGib1.2-hapl.c_protein.faa.gz
Pimephales promelas	GCF_016745375.1_EPA_FHM_2.0_protein.faa.gz
Labeo rohita	GCF_022985175.1_IGBB_LRoh.1.0_protein.faa.gz
Megalobrama amblycephala	GCF_018812025.1_ASM1881202v1_protein.faa.gz
Sinocyclocheilus grahami	GCF_001515645.1_SAMN03320097.WGS_v1.1_protein.faa.gz
Ctenopharyngodon idella	GCF_019924925.1_HZGC01_protein.faa.gz
Drosophila melanogaster	
Drosophila ananassae	GCF_017639315.1_ASM1763931v2_protein.faa.gz
Drosophila grimshawi	GCF_018153295.1_ASM1815329v1_protein.faa.gz
Drosophila pseudoobscura	GCF_009870125.1_UCI_Dpse_MV25_protein.faa.gz
Drosophila virilis	GCF_003285735.1_DvirRS2_protein.faa.gz
Drosophila willistoni	GCF_018902025.1_UCI_dwil_1.1_protein.faa.gz
Gallus gallus	
Lagopus muta	GCF_023343835.1_bLagMut1_primary_protein.faa.gz
Tympanuchus pallidicinctus	GCF_026119805.1_pur_lepc_1.0_protein.faa.gz
Lagopus leucura	GCF_019238085.1_USGS_WTPT01_protein.faa.gz
Centrocercus urophasianus	GCF_019232065.1_USGS_Curo_1.0_protein.faa.gz
Centrocercus urophasianus	GCF_019232065.1_USGS_Curo_1.0_protein.faa.gz
Coturnix japonica	GCF_001577835.2_Coturnix_japonica_2.1_protein.faa.gz

Meleagris gallopavo	GCF_000146605.3_Turkey_5.1_protein.faa.gz
Medicago truncatula	
Trifolium pratense	GCF_020283565.1_ARS_RC_1.1_protein.faa.gz
Pisum sativum	GCF_024323335.1_CAAS_Psat_ZW6_1.0_protein.faa.gz
Cicer arietinum	GCF_000331145.1_ASM33114v1_protein.faa.gz
Mus musculus	
Arvicanthis niloticus	GCF_011762505.1_mArvNil1.pat.X_protein.faa.gz
Grammomys surdaster	GCF_004785775.1_NIH_TR_1.0_protein.faa.gz
Mastomys coucha	GCF_008632895.1_UCSF_Mcou_1_protein.faa.gz
Mus pahari	GCF_900095145.1_PAHARI_EIJ_v1.1_protein.faa.gz
Apodemus sylvaticus	GCF_947179515.1_mApoSyl1.1_protein.faa.gz
Mus caroli	GCF_900094665.1_CAROLI_EIJ_v1.1_protein.faa.gz
Rattus rattus	GCF_011064425.1_Rrattus_CSIRO_v1_protein.faa.gz
Rattus norvegicus	GCF_015227675.2_mRatBN7.2_protein.faa.gz
Homo sapiens	GCF_000001405.40_GRCh38.p14_protein.faa.gz
Parasteatoda tepidariorum	
Trichonephila inaurata	GCA_019973955.1_Tnin_1.0_protein.faa.gz
Caerostris extrusa	GCA_021605095.1_Cext_1.0_protein.faa.gz
Caerostris darwini	GCA_021605075.1_Cdar_1.0_protein.faa.gz
Oedothorax gibbosus	GCA_019343175.1_Ogib_1.0_protein.faa.gz
Trichonephila clavata	GCA_019973975.1_Tnct_1.0_protein.faa.gz
Trichonephila clavipes	GCA_019973935.1_Tncv_1.0_protein.faa.gz
Araneus ventricosus	GCA_013235015.1_Ave_3.0_protein.faa.gz
Nephila pilipes	GCA_019974015.1_Npil_1.0_protein.faa.gz
Populus trichocarpa	
Populus tomentosa	GCA_018804465.1_PTv2_protein.faa.gz
Populus euphratica	GCF_000495115.1_PopEup_1.0_protein.faa.gz
Populus alba	GCF_005239225.1_ASM523922v1_protein.faa.gz
Populus deltoides	GCA_015852605.2_ASM1585260v2_protein.faa.gz
Solanum lycopersicum	
Solanum stenotomum	GCF_019186545.1_ASM1918654v1_protein.faa.gz
Solanum tuberosum	GCF_000226075.1_SolTub_3.0_protein.faa.gz
Solanum verrucosum	GCF_900185275.1_falcon-dt-bn_protein.faa.gz
Solanum pennellii	GCF_001406875.1_SPENNV200_protein.faa.gz

Supplemental Table S2: Donor proteins used for each species for the close relative included protein set.

Species	OrthoDB database	# BUSCO
Arabidopsis thaliana	eudicots_odb10	2,326
Bombus terrestris	hymenoptera_odb10	5,991
Caenorhabditis elegans	nematoda_odb10	3,131
Danio rerio	actinopterygii_odb10	3,640
Drosophila melanogaster	arthropoda_odb10	1,013
Gallus gallus	mammalia_odb10	9,226
Medicago truncatula	fabales_odb10	5,366
Mus musculus	mammalia_odb10	9,226
Parasteatoda tepidariorum	arachnida_odb10	2,934
Populus trichocarpa	eudicots_odb10	2,326
Prunus dulcis	viridiplantae_odb10	425
Solanum lycopersicum	solanales_odb10	5,950
Thrips palmi	insecta_odb10	1,367
Tetraselmis striata	chlorophyta_odb10	1,519

Supplemental Table S3: Summary of OrthoDB databases and number of BUSCOs used for BUSCO evaluation on each test species.

	Exon		Gene		Transcript		Exon		Gene		Transcript	
	Sn	Prec	Sn	Prec	Sn	Prec	Sn	Prec	Sn	Prec	Sn	Prec
	Arabidopsis thaliana						*Bombus terrestris*					
BRAKER1	78.20	80.83	58.37	58.73	39.97	56.75	78.86	69.77	42.02	27.45	28.72	25.67
BRAKER2	80.91	87.22	71.96	68.58	48.48	66.33	74.06	77.04	45.38	30.81	28.47	29.85
TSEBRA	71.02	93.68	70.92	84.38	49.26	75.30	65.61	79.95	56.87	44.26	39.24	38.17
GeneMark-ETP	**82.71**	89.67	76.59	78.22	53.11	76.76	**81.10**	85.64	62.26	58.36	45.16	54.20
BRAKER3	78.64	**95.81**	**77.90**	**86.53**	**54.25**	**82.30**	74.58	**89.64**	**66.82**	**64.51**	**49.19**	**56.66**
	Caenorhabditis elegans						*Danio rerio*					
BRAKER1	**82.00**	87.55	57.58	62.28	42.87	59.15	78.00	69.80	40.35	23.48	25.14	22.36
BRAKER2	74.13	87.97	47.76	54.30	33.87	53.11	76.14	68.71	41.30	20.12	25.11	19.37
TSEBRA	61.53	93.80	56.09	81.63	42.51	72.08	47.19	83.78	43.84	39.39	27.53	34.86
GeneMark-ETP	81.09	88.75	56.13	63.64	40.82	62.78	**78.12**	77.28	49.20	42.72	30.68	41.84
BRAKER3	65.89	**95.07**	**59.36**	**84.54**	**45.06**	**76.31**	61.89	**91.95**	**54.58**	**68.95**	**34.43**	**60.37**
	Drosophila melanogaster						*Gallus gallus*					
BRAKER1	76.81	76.77	59.58	58.69	39.40	55.26	63.28	46.92	5.96	2.97	5.09	2.88
BRAKER2	71.03	83.23	60.18	59.77	37.80	58.16	33.49	59.35	25.28	16.46	21.38	16.22
TSEBRA	65.72	89.99	69.18	79.69	46.26	70.45	26.85	77.34	25.61	27.11	21.77	26.03
GeneMark-ETP	**78.11**	89.78	72.68	77.85	48.77	75.61	**94.20**	87.49	76.41	61.06	68.87	58.24
BRAKER3	77.13	**94.46**	**78.71**	**87.73**	**54.03**	**79.96**	93.57	**94.79**	**84.11**	**79.73**	**75.25**	**70.05**
	Medicago truncatula						*Mus musculus*					
BRAKER1	76.77	65.93	36.95	37.59	36.95	35.78	82.22	66.24	28.01	13.09	28.00	12.70
BRAKER2	78.71	71.11	44.91	43.77	44.91	42.16	61.45	71.56	37.47	20.84	37.46	20.44
TSEBRA	66.50	85.33	46.01	62.73	46.01	53.97	51.58	79.74	42.11	30.90	42.10	27.99
GeneMark-ETP	**79.32**	79.62	45.86	63.62	45.86	59.80	**89.50**	88.14	67.47	57.85	67.47	56.05
BRAKER3	75.07	**90.31**	**46.96**	**75.47**	**46.96**	**67.94**	81.81	**96.73**	**75.84**	**83.05**	**75.83**	**73.11**
	Parasteatoda tepidariorum						*Populus trichocarpa*					
BRAKER1	**77.49**	63.48	41.65	22.80	34.25	21.45	81.13	72.11	50.56	44.31	40.52	42.29
BRAKER2	67.96	58.10	29.75	14.17	23.37	13.74	84.99	80.47	68.69	58.03	53.84	56.19
TSEBRA	48.34	73.59	44.65	31.98	37.08	29.11	74.72	90.31	68.66	76.85	55.30	66.53
GeneMark-ETP	76.52	72.39	45.56	41.66	38.99	40.97	**86.13**	88.69	72.88	78.47	58.95	74.49
BRAKER3	57.81	**87.61**	**47.16**	**61.51**	**40.69**	**55.47**	83.32	**95.10**	**74.90**	**88.76**	**60.68**	**81.63**
	Solanum lycopersicum						**Average**					
BRAKER1	75.24	62.44	33.09	29.39	33.09	27.62	77.27	69.26	41.28	34.62	32.18	32.90
BRAKER2	76.90	66.89	41.40	33.70	41.40	32.46	70.89	73.79	46.73	38.23	36.01	37.09
TSEBRA	68.74	79.93	43.94	50.13	43.94	42.17	58.89	84.31	51.63	55.37	41.00	48.79
GeneMark-ETP	**77.68**	80.15	43.65	55.75	43.65	51.25	**82.23**	84.33	60.79	61.75	49.30	59.27
BRAKER3	74.05	**86.85**	**44.46**	**63.66**	**44.46**	**56.51**	74.89	**92.57**	**64.62**	**76.77**	**52.80**	**69.12**

Supplemental Table S4: Sensitivity (Sn) and precision (Prec) for a protein database in which proteins of the same *order* as the target species were *excluded*. The last subtable shows the respective averages for the 11 different species. The highest number in each column is indicated in bold text. Inputs were for each species a genome assembly, short-read RNA-seq libraries, and a protein database (respective **order excluded**).

	Exon	Gene	Transcript	Exon	Gene	Transcript	Exon	Gene	Transcript	Exon	Gene	Transcript
	Arabidopsis thaliana			*Bombus terrestris*			*Caenorhabditis elegans*			*Danio rerio*		
BRAKER1	79.49	58.55	46.90	74.04	33.21	27.11	84.68	59.84	49.71	73.67	29.69	23.67
BRAKER2	83.95	70.23	56.02	75.52	36.70	29.14	80.46	50.82	41.36	72.23	27.06	21.87
TSEBRA	80.79	77.07	59.56	72.07	49.78	38.70	74.31	66.49	53.48	60.37	41.50	30.76
GeneMark-ETP	86.05	77.40	62.78	**83.31**	60.25	49.27	**84.75**	59.65	49.47	**77.70**	45.73	35.40
BRAKER3	**86.38**	**81.99**	**65.39**	81.42	**65.64**	**52.66**	77.84	**69.75**	**56.66**	73.98	**60.93**	**43.85**
	Drosophila melanogaster			*Gallus gallus*			*Medicago truncatula*			*Mus musculus*		
BRAKER1	76.79	59.13	46.00	53.89	3.96	3.68	70.94	37.27	36.36	73.37	17.84	17.47
BRAKER2	76.65	59.97	45.82	42.82	19.94	18.45	74.72	44.33	43.49	66.12	26.78	26.45
TSEBRA	75.96	74.06	55.85	39.86	26.34	23.71	74.75	53.08	49.67	62.64	35.64	33.62
GeneMark-ETP	83.54	75.18	59.29	90.72	67.88	63.11	79.47	53.30	51.91	**88.81**	62.29	61.23
BRAKER3	**84.92**	**82.98**	**64.49**	**94.18**	**81.86**	**72.56**	**81.99**	**57.90**	**55.53**	88.65	**79.28**	**74.45**
	Parasteatoda tepidariorum			*Populus trichocarpa*			*Solanum lycopersicum*			**Average**		
BRAKER1	69.79	29.47	26.38	76.35	47.23	41.39	68.24	31.13	30.11	73.05	37.66	32.54
BRAKER2	62.64	19.20	17.31	82.67	62.91	54.99	71.55	37.16	36.39	72.31	42.06	36.54
TSEBRA	58.35	37.27	32.62	81.78	72.52	60.40	73.91	46.83	43.04	69.35	53.43	44.56
GeneMark-ETP	**74.40**	43.52	39.96	87.39	75.57	65.82	78.90	48.96	47.15	**83.26**	61.26	53.83
BRAKER3	69.66	**53.39**	**46.94**	**88.82**	**81.24**	**69.61**	**79.94**	**52.36**	**49.77**	82.80	**70.17**	**59.87**

Supplemental Table S5: F1-scores of pipelines obtaining short-read RNA-seq libraries, and a protein database (respective **order excluded**) as input. The subtable on the bottom right shows the averages for the 11 different species. The highest number in each column is indicated in bold text.

	Exon		Gene		Transcript		Exon		Gene		Transcript	
	Sn	Prec	Sn	Prec	Sn	Prec	Sn	Prec	Sn	Prec	Sn	Prec
	Arabidopsis thaliana						*Bombus terrestris*					
BRAKER1	78.20	80.83	58.37	58.73	39.97	56.75	78.86	69.77	42.02	27.45	28.72	25.67
BRAKER2	83.20	86.83	79.18	72.88	53.81	69.00	81.29	75.63	61.9	35.57	39.90	33.96
TSEBRA	81.58	92.89	81.69	83.74	56.83	74.89	72.38	82.45	64.17	43.21	42.36	41.68
GeneMark-ETP	**83.45**	89.71	78.83	79.25	54.89	77.65	**83.37**	86.25	66.87	61.38	49.38	57.21
BRAKER3	81.72	**95.80**	**81.8**	**87.23**	**57.01**	**83.85**	78.56	**90.10**	**72.51**	**66.34**	**52.53**	**60.08**
	Caenorhabditis elegans						*Danio rerio*					
BRAKER1	82.00	87.55	57.58	62.28	42.87	59.15	78.00	69.80	40.35	23.48	25.14	22.36
BRAKER2	83.41	90.53	66.22	67.44	47.34	63.67	77.46	66.77	44.71	20.21	27.21	19.25
TSEBRA	76.22	93.85	**69.04**	82.25	**51.92**	71.96	60.70	82.37	53.19	39.6	33.34	34.8
GeneMark-ETP	**83.84**	90.07	63.98	69.45	47.73	67.38	**78.71**	76.58	50.27	41.58	31.34	40.77
BRAKER3	75.25	**95.45**	68.22	**85.54**	51.86	**77.64**	65.15	**90.79**	**56.91**	**66.60**	**35.83**	**58.23**
	Drosophila melanogaster						*Gallus gallus*					
BRAKER1	76.81	76.77	59.58	58.69	39.40	55.26	63.28	46.92	5.96	2.97	5.09	2.88
BRAKER2	79.64	88.45	77.78	73.76	49.63	70.36	36.16	61.33	28.56	19.31	24.25	18.93
TSEBRA	78.14	91.68	82.20	84.29	55.09	74.08	32.87	80.64	31.22	32.28	26.61	30.99
GeneMark-ETP	82.49	91.71	80.24	83.59	55.84	79.91	**94.14**	86.96	75.66	59.50	68.20	57.26
BRAKER3	**82.88**	**95.75**	**85.67**	**89.89**	**58.66**	**83.69**	93.92	**94.52**	**84.87**	**79.57**	**75.95**	**70.07**
	Medicago truncatula						*Mus musculus*					
BRAKER1	76.77	65.93	36.95	37.59	36.95	35.78	82.22	66.24	28.01	13.09	28.00	12.70
BRAKER2	79.84	71.47	47.09	46.56	47.09	43.98	61.70	71.34	40.04	22.10	40.03	21.54
TSEBRA	75.63	84.84	48.63	57.96	48.63	55.16	61.87	81.82	51.82	34.27	51.81	31.10
GeneMark-ETP	**80.16**	77.53	47.85	61.38	47.85	57.67	**90.15**	87.52	68.34	55.90	68.34	54.38
BRAKER3	76.89	**88.98**	**49.62**	**73.14**	**49.62**	**66.57**	85.37	**96.40**	**79.62**	**81.50**	**79.61**	**71.91**
	Parasteatoda tepidariorum						*Populus trichocarpa*					
BRAKER1	**77.49**	63.48	41.65	22.80	34.25	21.45	81.13	72.11	50.56	44.31	40.52	42.29
BRAKER2	69.11	58.97	32.24	15.06	25.33	14.46	86.45	80.58	73.36	61.11	57.90	58.52
TSEBRA	50.45	72.84	45.54	30.48	37.60	28.14	84.72	90.13	**77.75**	77.36	62.71	67.22
GeneMark-ETP	77.14	71.99	47.12	41.42	40.78	40.74	**86.65**	88.07	74.30	77.65	60.17	73.73
BRAKER3	58.53	**86.70**	**48.64**	**59.63**	**42.14**	**54.05**	85.08	**94.99**	77.75	**88.31**	**63.08**	**82.00**
	Solanum lycopersicum						**Average**					
BRAKER1	75.24	62.44	33.09	29.39	33.09	27.62	77.27	69.26	41.28	34.62	32.18	32.90
BRAKER2	78.43	60.74	44.59	31.55	44.59	29.25	74.24	73.88	54.15	42.32	41.55	40.27
TSEBRA	75.69	77.47	**48.46**	47.21	**48.46**	40.09	68.20	84.63	59.43	55.70	46.85	50.01
GeneMark-ETP	**78.59**	76.20	45.37	51.99	45.37	47.82	**83.52**	83.87	63.53	62.1	51.81	59.50
BRAKER3	75.87	**85.37**	46.90	**60.37**	46.90	**54.72**	78.11	**92.26**	**68.41**	**76.19**	**55.74**	**69.35**

Supplemental Table S6: Sensitivity (Sn) and precision (Prec) for a protein database in which only proteins from the target species were *excluded*. The last subtable shows the respective averages for the 11 different species. The highest number in each column is indicated in bold text. Inputs were for each species a genome assembly, short-read RNA-seq libraries, and a protein database (respective **species excluded**).

	Exon	Gene	Transcript	Exon	Gene	Transcript	Exon	Gene	Transcript	Exon	Gene	Transcript
	Arabidopsis thaliana			*Bombus terrestris*			*Caenorhabditis elegans*			*Danio rerio*		
BRAKER1	79.49	58.55	46.90	74.04	33.21	27.11	84.68	59.84	49.71	73.67	29.69	23.67
BRAKER2	84.98	75.90	60.47	78.36	45.18	36.69	86.82	66.82	54.30	71.72	27.84	22.55
TSEBRA	86.87	82.70	64.62	77.09	51.64	42.02	84.12	75.07	60.32	69.89	45.40	34.05
GeneMark-ETP	86.47	79.04	64.32	**84.79**	64.01	53.01	**86.84**	66.60	55.88	**77.63**	45.51	35.44
BRAKER3	**88.20**	**84.43**	**67.87**	83.94	**69.29**	**56.05**	84.15	**75.90**	**62.18**	75.86	**61.37**	**44.36**
	Drosophila melanogaster			*Gallus gallus*			*Medicago truncatula*			*Mus musculus*		
BRAKER1	76.79	59.13	46.00	53.89	3.96	3.68	70.94	37.27	36.36	73.37	17.84	17.47
BRAKER2	83.81	75.72	58.20	45.50	23.04	21.26	75.42	46.82	45.48	66.17	28.48	28.01
TSEBRA	84.37	83.23	63.19	46.70	31.74	28.63	79.97	52.89	51.69	70.46	41.26	38.87
GeneMark-ETP	86.86	81.88	65.74	90.41	66.61	62.25	78.82	53.78	52.30	88.82	61.50	60.57
BRAKER3	**88.85**	**87.73**	**68.97**	**94.22**	**82.13**	**72.89**	**82.49**	**59.13**	**56.86**	**90.55**	**80.55**	**75.56**
	Parasteatoda tepidariorum			*Populus trichocarpa*			*Solanum lycopersicum*			**Average**		
BRAKER1	69.79	29.47	26.38	76.35	47.23	41.39	68.24	31.13	30.11	73.05	37.66	32.54
BRAKER2	63.64	20.53	18.41	83.41	66.68	58.21	68.46	36.95	35.33	74.06	47.51	40.90
TSEBRA	59.61	36.52	32.19	87.34	77.55	64.89	76.57	47.83	43.88	75.54	57.50	48.38
GeneMark-ETP	**74.48**	44.09	40.76	87.35	75.94	66.26	77.38	48.45	46.56	83.69	62.81	55.39
BRAKER3	69.88	**53.58**	**47.36**	**89.76**	**82.69**	**71.31**	**80.34**	**52.79**	**50.51**	**84.60**	**72.09**	**61.81**

Supplemental Table S7: F1-scores of pipelines obtaining short-read RNA-seq libraries, and a protein database (respective **species excluded**) as input. The subtable on the bottom right shows the averages for the 11 different species. The highest number in each column is indicated in bold text.

	Exon			Gene			Transcript		
	Sn	Prec	F1	Sn	Prec	F1	Sn	Prec	F1
				Arabidopsis thaliana					
MAKER2*	78.05	82.02	79.99	60.57	57.69	59.09	40.72	57.69	47.74
Funannotate	82.07	93.21	87.29	75.26	79.58	77.36	50.59	79.58	61.86
BRAKER3	**83.03**	**94.38**	**88.34**	**82.93**	**86.27**	**84.57**	**58.70**	**80.78**	**67.99**
				Bombus terrestris					
MAKER2*	74.12	75.22	74.67	50.24	45.57	47.79	31.55	36.75	33.95
Funannotate	76.23	72.72	74.43	51.73	30.80	38.61	32.11	30.85	31.47
BRAKER3	**79.39**	**88.71**	**83.79**	**73.07**	**67.32**	**70.08**	**57.86**	**57.31**	**57.58**
				Caenorhabditis elegans					
MAKER2*	73.13	76.63	74.84	41.31	41.10	41.20	29.34	38.56	33.32
Funannotate	**79.71**	84.19	81.89	49.44	52.16	50.76	35.01	52.18	41.90
BRAKER3	76.39	**95.35**	**84.82**	**68.97**	**85.61**	**76.39**	**52.52**	**77.33**	**62.55**
				Drosophila melanogaster					
MAKER2*	75.28	73.11	74.18	61.10	52.77	56.63	38.26	52.77	44.36
Funannotate	73.87	82.66	78.02	62.87	62.98	62.92	39.38	62.99	48.46
BRAKER3	**81.64**	**95.18**	**87.89**	**83.36**	**90.55**	**86.81**	**59.71**	**81.58**	**68.95**
				Gallus gallus					
MAKER2*	85.98	78.83	82.25	49.42	38.65	43.38	41.99	32.64	36.73
Funannotate	58.75	71.22	64.39	32.23	23.20	26.98	27.19	23.23	25.05
BRAKER3	**93.82**	**93.99**	**93.90**	**85.80**	**78.88**	**82.19**	**78.39**	**66.65**	**72.04**
				Medicago truncatula					
MAKER2*	69.63	73.87	71.69	33.48	49.12	39.82	33.48	45.67	38.64
Funannotate	74.71	63.54	68.67	35.26	37.40	36.30	35.26	37.40	36.30
BRAKER3	**74.76**	**90.14**	**81.73**	**47.86**	**75.25**	**58.51**	**47.86**	**68.22**	**56.25**
				Populus trichocarpa					
MAKER2*	74.04	71.96	72.99	41.76	42.67	42.21	32.64	38.27	35.23
Funannotate	82.44	79.30	80.84	60.83	54.90	57.71	47.38	54.90	50.86
BRAKER3	**84.56**	**92.94**	**88.55**	**76.99**	**85.60**	**81.07**	**63.07**	**76.49**	**69.13**
				Solanum lycopersicum					
MAKER2*	74.16	45.21	56.17	33.88	18.85	24.22	33.88	18.85	24.22
Funannotate	74.62	65.91	70.00	36.12	34.10	35.08	36.12	34.10	35.08
BRAKER3	**74.87**	**85.25**	**79.72**	**45.90**	**61.67**	**52.63**	**45.90**	**53.49**	**49.41**

Supplemental Table S8: Sensitivity, precision, and F1-score for MAKER2, Funannotate, and BRAKER3 using the **close relatives included** protein databases. *The values for MAKER2 may overestimate the realistic performance on new genomes (see main text).

	Exon			Gene			Transcript		
	Sn	Prec	F1	Sn	Prec	F1	Sn	Prec	F1
Arabidopsis thaliana	18.0	50.42	26.53	6.03	18.42	9.09	4.16	16.68	6.66
Caenorhabditis elegans	28.62	50.67	36.58	8.10	12.4	9.80	5.89	11.54	7.80
Danio rerio	15.16	44.12	22.57	3.26	7.17	4.48	1.99	7.06	3.10
Drosophila melanogaster	24.28	53.95	33.49	11.32	23.01	15.17	7.48	21.18	11.06
Medicago truncatula	17.79	48.88	26.09	3.92	16.87	6.36	3.92	15.54	6.26
Populus trichocarpa	26.99	50.07	35.07	7.37	15.34	9.96	5.8	14.75	8.33
Solanum lycopersicum	31.21	43.98	36.51	6.41	12.68	8.52	6.41	10.12	7.85
**Average**	23.15	48.87	30.98	6.63	15.13	9.05	5.09	13.84	7.29

Supplemental Table S9: Sensitivity, precision, and F1-score of FINDER for runs using the same input data (genomic sequence + proteins + RNA-seq) as in the experiments from Supplemental Table S4. However, FINDER exited with an error for 4 out of the 11 species tested, and we therefore do not report its performance for those species.

	Exon			Gene			Transcript		
	Sn	Prec	F1	Sn	Prec	F1	Sn	Prec	F1
Arabidopsis thaliana	83.4	87.70	85.50	78.78	75.18	76.94	54.18	71.77	61.75
Bombus terrestris	84.49	84.17	84.33	67.38	55.01	60.57	46.56	50.28	48.35
Caenorhabditis elegans	84.97	91.99	88.34	70.36	74.46	72.35	51.99	70.34	59.79
Danio rerio	81.74	75.80	78.66	58.06	36.21	44.6	36.29	33.04	34.59
Drosophila melanogaster	83.49	89.93	86.59	80.87	80.71	80.79	54.09	75.66	63.08
Gallus gallus	96.17	89.11	92.51	81.70	61.03	69.87	71.73	57.34	63.73
Medicago truncatula	81.16	69.84	75.08	48.93	46.70	47.79	48.93	43.68	46.16
Mus musculus	94.51	85.48	89.77	79.46	46.23	58.45	79.46	43.42	56.15
Parasteatoda tepidariorum	78.67	65.55	71.51	48.13	26.32	34.03	39.77	24.53	30.34
Populus trichocarpa	87.70	81.99	84.75	75.77	63.60	69.15	60.80	60.97	60.88
Solanum lycopersicum	79.64	66.54	72.50	46.12	37.27	41.23	46.12	34.58	39.52
**Average**	85.09	80.74	82.69	66.87	54.79	59.62	53.63	51.42	51.30

Supplemental Table S10: Sensitivity, precision, and F1-score of the AUGUSTUS predictions made as part of the BRAKER3 pipeline. The results correspond to Supplemental Table S4 – proteins from the same order as the target species were excluded.

	Exon			Gene			Transcript		
	Sn	Prec	F1	Sn	Prec	F1	Sn	Prec	F1
Funannotate	73.20	74.40	73.79	47.11	44.69	45.87	35.75	44.70	39.73
Funannotate updated	73.20	74.40	73.79	47.11	44.69	45.87	35.75	44.70	39.73
Funannotate –repeats2evm	71.12	77.06	73.97	45.49	45.64	45.56	34.45	45.64	39.27
Funannotate –repeats2evm updated	73.20	74.40	73.79	47.11	44.69	45.87	35.75	44.70	39.73

Supplemental Table S11: Average sensitivity, precision, and F1-score for four different predictions generated by Funannotate using the **close relatives included** protein databases for the same species as listed in Supplemental Table S8. The prediction step of Funannotate was run with and without the option to pass gene predictions of repetitive regions to EVidenceModeler (`--repeats2evm`). The resulting predictions were both post-processed using Funannotate's update protocol, which updates the predicted gene models using RNA-seq data.

	Arabidopsis thaliana	*Bombus terrestris*	*Caenorhabditis elegans*	*Danio rerio*
BRAKER1	01:23	04:16	01:36	10:28
BRAKER2	06:39	06:27	04:57	19:47
GeneMark-ETP	04:05	02:56	03:36	09:54
BRAKER3	05:37	14:45	06:46	27:08
	Drosophila melanogaster	*Gallus gallus*	*Medicago truncatula*	*Mus musculus*
BRAKER1	01:55	10:12	03:20	19:41
BRAKER2	05:28	14:10	10:59	30:24
GeneMark-ETP	02:37	12:11	07:55	22:13
BRAKER3	06:50	42:44	13:14	64:16
	Parasteatoda tepidariorum	*Populus trichocarpa*	*Solanum lycopersicum*	**Average**
BRAKER1	14:47	04:41	03:47	06:55
BRAKER2	23:16	08:52	09:19	12:45
GeneMark-ETP	10:38	07:51	06:37	08:14
BRAKER3	35:35	11:41	12:06	21:53

Supplemental Table S12: Runtime of BRAKER1, BRAKER2, GeneMark-ETP, and BRAKER3 for all test species. The runtime is written as hours and minutes. The hardware is described in the caption of Supplemental Figure S6.

Species	Runtime (h:m)
Bombus terrestris	4:00
Drosophila melanogaster	2:06
Gallus gallus	13:54
Medicago truncatula	2:48
Populus trichocarpa	3:36

Supplemental Table S13: Runtime of MAKER2 with *close relatives included* database in MPI mode using LINUX nodes with 96 cores available through the Azure cloud. Training of *ab initio* gene finders and transcriptome assembly are not included in these figures.

	Arabidopsis thaliana	*Bombus terrestris*	*Caenorhabditis elegans*	*Drosophila melanogaster*
Funannotate	05:06	04:59	02:02	02:52
BRAKER3	05:46	14:38	04:13	06:25
	Gallus gallus	*Medicago truncatula*	*Populus trichocarpa*	*Solanum lycopersicum*
Funannotate	41:07	07:11	07:03	09:28
BRAKER3	27:58	07:54	08:19	08:25
	**Average**			
Funannotate	09:59			
BRAKER3	10:27			

Supplemental Table S14: Runtime of Funannotate and BRAKER3 for the experiments of Supplemental Table S8 (*close relatives included*). The runtime is written as hours and minutes. The hardware is described in the caption of Supplemental Figure S6.

Species	SRA ID	#spots	#bases	Date
A. tha.	SRR8714016	26,518,474	8,008,579,148	2020-02-27
	SRR8759751	21,388,481	6,458,011,735	2019-04-01
	SRR4010853	25,658,415	6,465,920,580	2017-05-10
	SRR7289569	9,222,308	1,391,511,444	2018-12-06
	SRR12547664	874,772	66,482,672	2021-04-07
	SRR12076896	21,857,417	6,557,225,100	2021-05-24
B. ter.	SRR5125126	28,930,495	5,207,489,100	2017-10-30
	SRR5125123	25,185,030	4,533,305,400	2017-10-30
	SRR16931591	25,056,341	7,516,902,300	2021-11-12
	SRR5125133	26,059,038	4,690,626,840	2017-10-30
	SRR5125134	23,745,975	4,274,275,500	2017-10-30
	SRR8085469	32,768,117	6,553,623,400	2019-03-05
C. ele.	SRR7446944	44,853,824	8,970,764,800	2020-06-01
	ERR2756716	4,785,656	1,445,268,112	2018-08-29
	SRR6815567	5,901,194	890,097,086	2018-03-08
	SRR6474814	34,199,521	8,618,279,292	2018-01-16
	SRR10238291	47,652,843	4,955,895,672	2019-11-07
D. rer.	SRR3179613	9,994,443	2,018,877,486	2017-02-10
	ERR1857957	2,536,494	380,474,100	2017-03-03
	SRR9159941	19,527,035	5,858,110,500	2019-12-01
	SRR9159937	37,156,875	11,147,062,500	2019-12-01
	ERR958944	10,490,607	1,363,778,910	2015-07-17
	SRR8106574	1,281,189	186,371,148	2020-01-20
D. mel.	SRR19416937	21,377,196	6,413,158,800	2022-05-30
	SRR19416947	22,611,252	6,783,375,600	2022-05-30
	SRR19416944	17,754,393	5,326,317,900	2022-05-30
	SRR19446462	40,819,492	4,163,588,184	2022-05-30
	SRR19416948	21,257,788	6,377,336,400	2022-05-30
M. tru.	SRR3735569	13,021,000	2,630,242,000	2016-07-02
	SRR2016009	4,289,979	428,997,900	2016-05-12
	SRR10416790	9,757,388	2,927,216,400	2019-11-23
	SRR3726824	44,634,145	13,390,243,500	2016-06-28
	SRR2015998	7,916,226	791,622,600	2016-05-12
	SRR15462191	21,367,770	6,410,331,000	2021-08-15
P. tep.	SRR5458595	94,367,928	18,873,585,600	2017-06-14
	SRR1824488	33,099,205	6,686,039,410	2015-03-03
	SRR12687629	41,410,278	12,423,083,400	2021-06-22
	SRR8755634	45,919,505	8,852,599,087	2019-03-20
	SRR5602551	9,600,853	2,400,213,250	2017-10-16
	SRR1824489	60,177,256	12,155,805,712	2015-03-03
P. tri.	SRR3019959	32,156,743	4,591,697,381	2016-01-19
	SRR3019957	44,863,324	6,484,994,933	2016-01-19
	SRR3019251	29,773,370	4,305,817,693	2016-01-19
	SRR3019585	36,843,662	5,279,588,065	2016-01-19
	SRR3019304	41,358,002	5,970,634,746	2016-01-19
	SRR12671667	17,429,154	5,263,604,508	2020-09-18
G. gal.	SRR5340686	27,956,970	2,851,610,940	2017-03-15
	ERR2113192	20,988,313	3,148,246,950	2017-09-07
	ERR2113173	21,562,175	3,234,326,250	2017-09-07
	SRR5190436	23,938,529	4,787,705,800	2017-09-06
	SRR1822373	48,874,648	7,819,943,680	2015-05-05
	SRR5437696	35,378,254	5,306,738,100	2017-09-18
M. mus.	SRR5197958	10,577,301	3,184,479,151	2017-08-31
	SRR3094250	1,568,252	316,786,904	2016-11-14
	SRR6067921	5,886,768	888,143,922	2018-08-03
	SRR10115888	44,307,998	8,861,599,600	2020-10-02
	ERR3005082	8,598	1,289,700	2019-01-02
	SRR9202226	5,355,073	546,217,446	2019-08-07

Supplemental Table S15: This table lists all RNA-seq libraries used for each species in all experiments. It includes the ID of the library at the Sequence Read Archive, the number of spots, number of bases, and publication date.

Species	AUGUSTUS	GeneMark.hmm	SNAP
A. thaliana	arabidopsis*	from GeneMark-ES run	A.thaliana.hmm*
B. terrestris	bombus_terrestris2*	from GeneMark-EP+ run	trained with genes from ref. annotation
C. elegans	c_elegans_trsk*	from GeneMark-ES run	Ce.hmm*
D. melanogaster	fly*	from GeneMark-ES run	fly*
G. gallus	chicken*	medium GC model from GeneMark-ETP run	trained with genes from ref. annotation
M. truncatula	trained with genes from ref. annotation	from GeneMark-EP+ run	trained with genes from ref. annotation
P. trichocarpa	trained with genes from ref. annotation	from GeneMark-EP+ run	trained with genes from ref. annotation
S. lycopersicum	tomato*	from GeneMark-EP+ run	trained with genes from ref. annotation

Supplemental Table S16: Configuration of AUGUSTUS, GeneMark.hmm and SNAP gene models using parameter from the AUGUSTUS and the SNAP distribution packages (marked with *) or with manually training using a training gene set.

1.3 Supplementary Methods

Running BRAKER

BRAKER v3.0.2 was installed from GitHub (`https://github.com/Gaius-Augustus/BRAKER`) and the BRAKER pipelines were run as follows. The GeneMark-ETP results were taken from the BRAKER3 runs:

BRAKER1:

```
braker.pl --genome=genome.softmasked.fasta --threads=48 \
    --rnaseq_sets_dirs=/location/of/local/RNA_Seq/Files \
    --rnaseq_sets_ids=RNA_Seq_IDS
```

BRAKER2:

```
braker.pl --genome=genome.softmasked.fasta --prot_seq=proteins.fa \
     --threads=48
```

BRAKER3:

```
braker.pl --genome=genome.softmasked.fasta --prot_seq=proteins.fa \
    --rnaseq_sets_dirs=/location/of/local/RNA_Seq/Files \
    --rnaseq_sets_ids=RNA_Seq_IDS --threads=48
```

Running Funannotate

Funannotate v1.8.14 was installed using a Singularity container as follows:

```
# only once, to get the singularity container
singularity pull docker://nextgenusfs/funannotate

export GENEMARK_PATH=/path/to/GeneMark-ES-ET-EP_v4.71_lic

species="name of species"
buscoSeedSpecies="name of seed species"
buscodb="name of busco db"
genome="/path/to/genome.fasta.masked"
proteins="/path/to/proteins.fa"
bamfile="/path/to/bamfile.bam"

# calculateGenomeSizeFromFasta.pl adds up the length of all sequences in a fasta
genomeSize=$(perl ~/calculateGenomeSizeFromFasta.pl $genome)
maxIntronLen_f=$(echo "3.6 * sqrt($genomeSize)" | bc -l)
maxIntronLen=$(printf "%.0f" "$maxIntronLen_f")

singularity run funannotate_latest.sif funannotate sort \
    --input $genome \
    --out genome.sorted.fa \
    --simplify
```

We produced four different results for each species, using the option `--repeats2evm` during the prediction step and a recommended update step:

```
mkdir -p fun tmp
# run prediction
singularity run funannotate_latest.sif funannotate predict \
    --input genome.sorted.fa --out fun --species $species \
    --busco_seed_species $buscoSeedSpecies --busco_db $buscodb \
    --organism other --protein_evidence $proteins \
    --rna_bam $bamfile --max_intronlen $maxIntronLen \
    --cpus 72 --tmpdir tmp --no-progress \
    [--repeats2evm]
```

```
# run update step
singularity run funannotate_latest.sif funannotate update \
  --input fun/ --left $readL --right $readR \
  --max_intronlen $maxIntronLen --species $species \
  --memory 50G --cpus 72 --no-progress
```

Running FINDER

FINDER v1.1.0 was installed from GitHub (`https://github.com/sagnikbanerjee15/Finder`) and run as follows:

```
run_finder --protein proteins.fa --framework singularity --output_directory finder --cpu 48 \
    --metadatafile metadata.csv --genome genome.softmasked.fasta --genemark_license gm_key \
    --genemark_path /location/of/GeneMark-ES/ET/EP  --organism_model {VERT,INV,PLANTS,FUNGI}
```

The information in the `metadata.csv` files was manually generated for each species and includes details about the RNA-seq libraries. The files consist of the following fields:

```
BioProject, SRA Accession, Tissues, Description, Date, Read Length (bp),
Ended, RNA Seq, process, Location
```

Running MAKER2

We ran MAKER2 version 3.01.04 using AUGUSTUS version 3.5.0, GeneMark.hmm version 3.68, SNAP version 2.59.5, Exonerate version 2.2.0, BLAST version 2.14.0, Tandem Repeats Finder version 4.09.

We provided MAKER with a GFF file containing the coordinates of repeats, as well as their predicted repeat types from RepeatMasker. To ensure compatibility with MAKER, we reformatted the RepeatMasker output to a specific GFF format using the following script from `https://github.com/gatech-genemark/BRAKER2-exp.git` and command line:

```
rmasker_out2maker_gff.pl < genome.fasta.out > rmasker4maker.gff
```

This script is available from the repository `https://github.com/gatech-genemark/GeneMark-ETP-exp`.

The default MAKER2 configuration was adjusted with following settings:

```
genome=genome.fasta
est=transcriptome.fasta
protein=proteindb.fasta
model_org=   #empty
rm_gff=repeatmasker.gff
snaphmm=snap.model
gmhmm=genemark.mod
augustus_species=model_name
est2genome=1
protein2genome=1
keep_preds=0
```

Here, `transcriptome.fasta` contained the same transcriptome assemblies that were used with BRAKER3 and constructed with HISAT2 and StringTie2. MAKER2 was then run with:

```
mpiexec.mpich -n 96  maker
```

Preparing protein data

OrthoDB v.11 was partitioned into proteins of species from the clade *Arthropoda*, *Metazoa*, *Vertebrata*, and *Viridiplantae*. The partitioning is available from `https://bioinf.uni-greifswald.de/bioinf/partitioned_odb11/`. Subsequently, two protein sets for each species from Suppl. Table 1 were generated, excluding either only the target species (species-excluded) or all species of the same taxonomic order (order-excluded).

These sets were prepared using the orthodb-clades pipeline, downloaded from GitHub (`https://github.com/tomasbruna/orthodb-clades`).

```
snakemake --cores 48
```

Accuracy evaluation

The performance measurements were computed using scripts from the BRAKER suite:

```
compute_accuracies.sh ref_annot.gtf pseudo.gff3 gene_set.gtf gene trans cds
```

Measuring expression

Expression levels were estimated with `kallisto` version 0.50.0 (Bray et al., 2016). For each species, the mRNA sequences of the respective reference annotation were quantified using a pool of RNA-seq libraries. More specifically, all paired libraries that were used for annotation were pooled and TPM (transcript per million) values estimated with the following command line.

```
kallisto quant -i kallisto.idx -o work/ -t 8 --verbose input/*.fastq
```

Subsequently, the transcripts for each species were partitioned in three expression *terciles*: the first with lowest TPM value, the second with medium TPM value and the third with highest TPM value.
