# Supplementary material for: BRAKER3: Fully Automated Genome Annotation Using RNA-Seq and Protein Evidence with GeneMark-ETP, AUGUSTUS and TSEBRA
Source: bioRxiv. 2023 Sep 2:2023.06.10.544449. Originally published 2023 Jun 12. Preprint. [Version 2] doi: 10.1101/2023.06.10.544449 (PMC10312602; doi:10.1101/2023.06.10.544449)
Supplement: Supplementary file 1 [file media-1.pdf]

1 Supplementary Information

1.1 Supplementary Tables

Species	Size (Mbp)	#Genes	#Transcripts	#CDS	# Sequences in protein database		
					species excluded	*order excluded*	*close relatives included*
Arabidopsis thaliana	119	27,445	48,149	156,731	5,283,100	4,825,128	306,719
Bombus terrestris	249	10,581	22,091	78,337	4,297,173	3,416,393	180,811
Caenorhabditis elegans	100	20,172	33,624	130,885	15,237,008	15,145,380	115,553
Danio rerio	1,345	25,611	42,934	262,325	9,779,764	9,468,332	760,754
Drosophila melanogaster	138	13,930	30,561	62,841	4,293,925	3,029,616	116,493
Gallus gallus	1,050	17,279	38,534	192,095	9,787,814	9,690,812	220,641
Medicago truncatula	420	44,464	44,464	174,281	5,278,627	4,680,430	134,774
Mus musculus	2,723	22,405	58,318	243,366	9,782,804	9,059,968	510,476
Parasteatoda tepidariorum	1,445	18,602	27,516	143,792	4,287,893	4,240,214	1,163,197
Populus trichocarpa	389	34,488	52,085	187,170	5,278,879	5,112,927	199,662
Solanum lycopersicum	773	33,562	33,562	152,352	5,284,979	4,823,899	149,444

Table 1: Summary of genomes, annotations and protein databases used for accuracy evaluation. Data extracted from Table 4 in Brůna et al. [2023] and computed from raw data of Brůna et al. [2021, 2023]. Genome versions, repeat masking and annotation processing are documented at `https://github.com/gatech-genemark/EukSpecies-BRAKER2` and at `https://github.com/gatech-genemark/GeneMark-ETP-exp`. The protein databases were generated with orthodb-clades from `https://github.com/tomasbruna/orthodb-clades`.

	Gene		Transcript		Exon		Gene		Transcript		Exon	
	Sn	Sp	Sn	Sp	Sn	Sp	Sn	Sp	Sn	Sp	Sn	Sp
	Arabidopsis thaliana						*Bombus terrestris*					
BRAKER1	58.37	58.73	39.97	56.75	78.20	80.83	42.02	27.45	28.72	25.67	78.86	69.77
BRAKER2	71.96	68.58	48.48	66.33	80.91	87.22	45.38	30.81	28.47	29.85	74.06	77.04
TSEBRA	70.92	84.38	49.26	75.30	71.02	93.68	56.87	44.26	39.24	38.17	65.61	79.95
GeneMark-ETP	76.59	78.22	53.11	76.76	**82.71**	89.67	62.26	58.36	45.16	54.20	**81.10**	85.64
BRAKER3	**77.90**	**86.53**	**54.25**	**82.30**	78.64	**95.81**	**66.82**	**64.51**	**49.19**	**56.66**	74.58	**89.64**
	Caenorhabditis elegans						*Danio rerio*					
BRAKER1	57.58	62.28	42.87	59.15	**82.00**	87.55	40.35	23.48	25.14	22.36	78.00	69.80
BRAKER2	47.76	54.30	33.87	53.11	74.13	87.97	41.30	20.12	25.11	19.37	76.14	68.71
TSEBRA	56.09	81.63	42.51	72.08	61.53	93.80	43.84	39.39	27.53	34.86	47.19	83.78
GeneMark-ETP	56.13	63.64	40.82	62.78	81.09	88.75	49.20	42.72	30.68	41.84	**78.12**	77.28
BRAKER3	**59.36**	**84.54**	**45.06**	**76.31**	65.89	**95.07**	**54.58**	**68.95**	**34.43**	**60.37**	61.89	**91.95**
	Drosophila melanogaster						*Gallus gallus*					
BRAKER1	59.58	58.69	39.40	55.26	76.81	76.77	5.96	2.97	5.09	2.88	63.28	46.92
BRAKER2	60.18	59.77	37.80	58.16	71.03	83.23	25.28	16.46	21.38	16.22	33.49	59.35
TSEBRA	69.18	79.69	46.26	70.45	65.72	89.99	25.61	27.11	21.77	26.03	26.85	77.34
GeneMark-ETP	72.68	77.85	48.77	75.61	**78.11**	89.78	76.41	61.06	68.87	58.24	**94.20**	87.49
BRAKER3	**78.71**	**87.73**	**54.03**	**79.96**	77.13	**94.46**	**84.11**	**79.73**	**75.25**	**70.05**	93.57	**94.79**
	Medicago truncatula						*Mus musculus*					
BRAKER1	36.95	37.59	36.95	35.78	76.77	65.93	28.01	13.09	28.00	12.70	82.22	66.24
BRAKER2	44.91	43.77	44.91	42.16	78.71	71.11	37.47	20.84	37.46	20.44	61.45	71.56
TSEBRA	46.01	62.73	46.01	53.97	66.50	85.33	42.11	30.90	42.10	27.99	51.58	79.74
GeneMark-ETP	45.86	63.62	45.86	59.80	**79.32**	79.62	67.47	57.85	67.47	56.05	**89.50**	88.14
BRAKER3	**46.96**	**75.47**	**46.96**	**67.94**	75.07	**90.31**	**75.84**	**83.05**	**75.83**	**73.11**	81.81	**96.73**
	Parasteatoda tepidariorum						*Populus trichocarpa*					
BRAKER1	41.65	22.80	34.25	21.45	**77.49**	63.48	50.56	44.31	40.52	42.29	81.13	72.11
BRAKER2	29.75	14.17	23.37	13.74	67.96	58.10	68.69	58.03	53.84	56.19	84.99	80.47
TSEBRA	44.65	31.98	37.08	29.11	48.34	73.59	68.66	76.85	55.30	66.53	74.72	90.31
GeneMark-ETP	45.56	41.66	38.99	40.97	76.52	72.39	72.88	78.47	58.95	74.49	**86.13**	88.69
BRAKER3	**47.16**	**61.51**	**40.69**	**55.47**	57.81	**87.61**	**74.90**	**88.76**	**60.68**	**81.63**	83.32	**95.10**
	Solanum lycopersicum						**Average**					
BRAKER1	33.09	29.39	33.09	27.62	75.24	62.44	41.28	34.62	32.18	32.90	77.27	69.26
BRAKER2	41.40	33.70	41.40	32.46	76.90	66.89	46.73	38.23	36.01	37.09	70.89	73.79
TSEBRA	43.94	50.13	43.94	42.17	68.74	79.93	51.63	55.37	41.00	48.79	58.89	84.31
GeneMark-ETP	43.65	55.75	43.65	51.25	**77.68**	80.15	60.79	61.75	49.30	59.27	**82.23**	84.33
BRAKER3	**44.46**	**63.66**	**44.46**	**56.51**	74.05	**86.85**	**64.62**	**76.77**	**52.80**	**69.12**	74.89	**92.57**

Table 2: Sensitivity (Sn) and specificity (Sp) for a protein database in which proteins of the same *order* as the target species were *excluded*. The last subtable shows the respective averages for the 11 different species. The highest number in each column is indicated in bold text. Inputs were for each species a genome assembly, short-read RNA-Seq libraries, and a protein database (respective **order excluded**).

	Gene	Transcript	Exon	Gene	Transcript	Exon	Gene	Transcript	Exon	Gene	Transcript	Exon
	Arabidopsis thaliana			*Bombus terrestris*			*Caenorhabditis elegans*			*Danio rerio*		
BRAKER1	58.55	46.90	79.49	33.21	27.11	74.04	59.84	49.71	84.68	29.69	23.67	73.67
BRAKER2	70.23	56.02	83.95	36.70	29.14	75.52	50.82	41.36	80.46	27.06	21.87	72.23
TSEBRA	77.07	59.56	80.79	49.78	38.70	72.07	66.49	53.48	74.31	41.50	30.76	60.37
GeneMark-ETP	77.40	62.78	86.05	60.25	49.27	**83.31**	59.65	49.47	**84.75**	45.73	35.40	**77.70**
BRAKER3	**81.99**	**65.39**	**86.38**	**65.64**	**52.66**	81.42	**69.75**	**56.66**	77.84	**60.93**	**43.85**	73.98
	Drosophila melanogaster			*Gallus gallus*			*Medicago truncatula*			*Mus musculus*		
BRAKER1	59.13	46.00	76.79	3.96	3.68	53.89	37.27	36.36	70.94	17.84	17.47	73.37
BRAKER2	59.97	45.82	76.65	19.94	18.45	42.82	44.33	43.49	74.72	26.78	26.45	66.12
TSEBRA	74.06	55.85	75.96	26.34	23.71	39.86	53.08	49.67	74.75	35.64	33.62	62.64
GeneMark-ETP	75.18	59.29	83.54	67.88	63.11	90.72	53.30	51.91	79.47	62.29	61.23	**88.81**
BRAKER3	**82.98**	**64.49**	**84.92**	**81.86**	**72.56**	**94.18**	**57.90**	**55.53**	**81.99**	**79.28**	**74.45**	88.65
	Parasteatoda tepidariorum			*Populus trichocarpa*			*Solanum lycopersicum*			**Average**		
BRAKER1	29.47	26.38	69.79	47.23	41.39	76.35	31.13	30.11	68.24	37.66	32.54	73.05
BRAKER2	19.20	17.31	62.64	62.91	54.99	82.67	37.16	36.39	71.55	42.06	36.54	72.31
TSEBRA	37.27	32.62	58.35	72.52	60.40	81.78	46.83	43.04	73.91	53.43	44.56	69.35
GeneMark-ETP	43.52	39.96	**74.40**	75.57	65.82	87.39	48.96	47.15	78.90	61.26	53.83	**83.26**
BRAKER3	**53.39**	**46.94**	69.66	**81.24**	**69.61**	**88.82**	**52.36**	**49.77**	**79.94**	**70.17**	**59.87**	82.80

Table 3: F1-scores of pipelines obtaining short-read RNA-Seq libraries, and a protein database (respective **order excluded**) as input. The subtable on the bottom right shows the averages for the 11 different species. The highest number in each column is indicated in bold text.

	Gene		Transcript		Exon		Gene		Transcript		Exon	
	Sn	Sp	Sn	Sp	Sn	Sp	Sn	Sp	Sn	Sp	Sn	Sp
	Arabidopsis thaliana						*Bombus terrestris*					
BRAKER1	58.37	58.73	39.97	56.75	78.20	80.83	42.02	27.45	28.72	25.67	78.86	69.77
BRAKER2	79.18	72.88	53.81	69.00	83.20	86.83	61.9	35.57	39.90	33.96	81.29	75.63
TSEBRA	81.69	83.74	56.83	74.89	81.58	92.89	64.17	43.21	42.36	41.68	72.38	82.45
GeneMark-ETP	78.83	79.25	54.89	77.65	**83.45**	89.71	66.87	61.38	49.38	57.21	**83.37**	86.25
BRAKER3	**81.8**	**87.23**	**57.01**	**83.85**	81.72	**95.80**	**72.51**	**66.34**	**52.53**	**60.08**	78.56	**90.10**
	Caenorhabditis elegans						*Danio rerio*					
BRAKER1	57.58	62.28	42.87	59.15	82.00	87.55	40.35	23.48	25.14	22.36	78.00	69.80
BRAKER2	66.22	67.44	47.34	63.67	83.41	90.53	44.71	20.21	27.21	19.25	77.46	66.77
TSEBRA	**69.04**	82.25	**51.92**	71.96	76.22	93.85	53.19	39.6	33.34	34.8	60.70	82.37
GeneMark-ETP	63.98	69.45	47.73	67.38	**83.84**	90.07	50.27	41.58	31.34	40.77	**78.71**	76.58
BRAKER3	68.22	**85.54**	51.86	**77.64**	75.25	**95.45**	**56.91**	**66.60**	**35.83**	**58.23**	65.15	**90.79**
	Drosophila melanogaster						*Gallus gallus*					
BRAKER1	59.58	58.69	39.40	55.26	76.81	76.77	5.96	2.97	5.09	2.88	63.28	46.92
BRAKER2	77.78	73.76	49.63	70.36	79.64	88.45	28.56	19.31	24.25	18.93	36.16	61.33
TSEBRA	82.20	84.29	55.09	74.08	78.14	91.68	31.22	32.28	26.61	30.99	32.87	80.64
GeneMark-ETP	80.24	83.59	55.84	79.91	82.49	91.71	75.66	59.50	68.20	57.26	**94.14**	86.96
BRAKER3	**85.67**	**89.89**	**58.66**	**83.69**	**82.88**	**95.75**	**84.87**	**79.57**	**75.95**	**70.07**	93.92	**94.52**
	Medicago truncatula						*Mus musculus*					
BRAKER1	36.95	37.59	36.95	35.78	76.77	65.93	28.01	13.09	28.00	12.70	82.22	66.24
BRAKER2	47.09	46.56	47.09	43.98	79.84	71.47	40.04	22.10	40.03	21.54	61.70	71.34
TSEBRA	48.63	57.96	48.63	55.16	75.63	84.84	51.82	34.27	51.81	31.10	61.87	81.82
GeneMark-ETP	47.85	61.38	47.85	57.67	**80.16**	77.53	68.34	55.90	68.34	54.38	**90.15**	87.52
BRAKER3	**49.62**	**73.14**	**49.62**	**66.57**	76.89	**88.98**	**79.62**	**81.50**	**79.61**	**71.91**	85.37	**96.40**
	Parasteatoda tepidariorum						*Populus trichocarpa*					
BRAKER1	41.65	22.80	34.25	21.45	**77.49**	63.48	50.56	44.31	40.52	42.29	81.13	72.11
BRAKER2	32.24	15.06	25.33	14.46	69.11	58.97	73.36	61.11	57.90	58.52	86.45	80.58
TSEBRA	45.54	30.48	37.60	28.14	50.45	72.84	**77.75**	77.36	62.71	67.22	84.72	90.13
GeneMark-ETP	47.12	41.42	40.78	40.74	77.14	71.99	74.30	77.65	60.17	73.73	**86.65**	88.07
BRAKER3	**48.64**	**59.63**	**42.14**	**54.05**	58.53	**86.70**	77.75	**88.31**	**63.08**	**82.00**	85.08	**94.99**
	Solanum lycopersicum						**Average**					
BRAKER1	33.09	29.39	33.09	27.62	75.24	62.44	41.28	34.62	32.18	32.90	77.27	69.26
BRAKER2	44.59	31.55	44.59	29.25	78.43	60.74	54.15	42.32	41.55	40.27	74.24	73.88
TSEBRA	**48.46**	47.21	**48.46**	40.09	75.69	77.47	59.43	55.70	46.85	50.01	68.20	84.63
GeneMark-ETP	45.37	51.99	45.37	47.82	**78.59**	76.20	63.53	62.1	51.81	59.50	**83.52**	83.87
BRAKER3	46.90	**60.37**	46.90	**54.72**	75.87	**85.37**	**68.41**	**76.19**	**55.74**	**69.35**	78.11	**92.26**

Table 4: Sensitivity (Sn) and specificity (Sp) for a protein database in which only proteins from the target species were *excluded*. The last subtable shows the respective averages for the 11 different species. The highest number in each column is indicated in bold text. Inputs were for each species a genome assembly, short-read RNA-Seq libraries, and a protein database (respective **species excluded**).

	Gene	Transcript	Exon	Gene	Transcript	Exon	Gene	Transcript	Exon	Gene	Transcript	Exon
	Arabidopsis thaliana			*Bombus terrestris*			*Caenorhabditis elegans*			*Danio rerio*		
BRAKER1	58.55	46.90	79.49	33.21	27.11	74.04	59.84	49.71	84.68	29.69	23.67	73.67
BRAKER2	75.90	60.47	84.98	45.18	36.69	78.36	66.82	54.30	86.82	27.84	22.55	71.72
TSEBRA	82.70	64.62	86.87	51.64	42.02	77.09	75.07	60.32	84.12	45.40	34.05	69.89
GeneMark-ETP	79.04	64.32	86.47	64.01	53.01	**84.79**	66.60	55.88	**86.84**	45.51	35.44	**77.63**
BRAKER3	**84.43**	**67.87**	**88.20**	**69.29**	**56.05**	83.94	**75.90**	**62.18**	84.15	**61.37**	**44.36**	75.86
	Drosophila melanogaster			*Gallus gallus*			*Medicago truncatula*			*Mus musculus*		
BRAKER1	59.13	46.00	76.79	3.96	3.68	53.89	37.27	36.36	70.94	17.84	17.47	73.37
BRAKER2	75.72	58.20	83.81	23.04	21.26	45.50	46.82	45.48	75.42	28.48	28.01	66.17
TSEBRA	83.23	63.19	84.37	31.74	28.63	46.70	52.89	51.69	79.97	41.26	38.87	70.46
GeneMark-ETP	81.88	65.74	86.86	66.61	62.25	90.41	53.78	52.30	78.82	61.50	60.57	88.82
BRAKER3	**87.73**	**68.97**	**88.85**	**82.13**	**72.89**	**94.22**	**59.13**	**56.86**	**82.49**	**80.55**	**75.56**	**90.55**
	Parasteatoda tepidariorum			*Populus trichocarpa*			*Solanum lycopersicum*			**Average**		
BRAKER1	29.47	26.38	69.79	47.23	41.39	76.35	31.13	30.11	68.24	37.66	32.54	73.05
BRAKER2	20.53	18.41	63.64	66.68	58.21	83.41	36.95	35.33	68.46	47.51	40.90	74.06
TSEBRA	36.52	32.19	59.61	77.55	64.89	87.34	47.83	43.88	76.57	57.50	48.38	75.54
GeneMark-ETP	44.09	40.76	**74.48**	75.94	66.26	87.35	48.45	46.56	77.38	62.81	55.39	83.69
BRAKER3	**53.58**	**47.36**	69.88	**82.69**	**71.31**	**89.76**	**52.79**	**50.51**	**80.34**	**72.09**	**61.81**	**84.60**

Table 5: F1-scores of pipelines obtaining short-read RNA-Seq libraries, and a protein database (respective **species excluded**) as input. The subtable on the bottom right shows the averages for the 11 different species. The highest number in each column is indicated in bold text.

	Gene			Transcript			Exon		
	Sn	Sp	F1	Sn	Sp	F1	Sn	Sp	F1
				Arabidopsis thaliana					
MAKER2*	60.57	57.69	59.09	40.72	57.69	47.74	78.05	82.02	79.99
Funannotate	75.26	79.58	77.36	50.59	79.58	61.86	82.07	93.21	87.29
BRAKER3	**82.93**	**86.27**	**84.57**	**58.70**	**80.78**	**67.99**	**83.03**	**94.38**	**88.34**
				Bombus terrestris					
MAKER2*	50.24	45.57	47.79	31.55	36.75	33.95	74.12	75.22	74.67
Funannotate	51.73	30.80	38.61	32.11	30.85	31.47	76.23	72.72	74.43
BRAKER3	**73.07**	**67.32**	**70.08**	**57.86**	**57.31**	**57.58**	**79.39**	**88.71**	**83.79**
				Caenorhabditis elegans					
MAKER2*	41.31	41.10	41.20	29.34	38.56	33.32	73.13	76.63	74.84
Funannotate	49.44	52.16	50.76	35.01	52.18	41.90	**79.71**	84.19	81.89
BRAKER3	**68.97**	**85.61**	**76.39**	**52.52**	**77.33**	**62.55**	76.39	**95.35**	**84.82**
				Drosophila melanogaster					
MAKER2*	61.10	52.77	56.63	38.26	52.77	44.36	75.28	73.11	74.18
Funannotate	62.87	62.98	62.92	39.38	62.99	48.46	73.87	82.66	78.02
BRAKER3	**83.36**	**90.55**	**86.81**	**59.71**	**81.58**	**68.95**	**81.64**	**95.18**	**87.89**
				Gallus gallus					
MAKER2*	49.42	38.65	43.38	41.99	32.64	36.73	85.98	78.83	82.25
Funannotate	32.23	23.20	26.98	27.19	23.23	25.05	58.75	71.22	64.39
BRAKER3	**85.80**	**78.88**	**82.19**	**78.39**	**66.65**	**72.04**	**93.82**	**93.99**	**93.90**
				Medicago truncatula					
MAKER2*	33.48	49.12	39.82	33.48	45.67	38.64	69.63	73.87	71.69
Funannotate	35.26	37.40	36.30	35.26	37.40	36.30	74.71	63.54	68.67
BRAKER3	**47.86**	**75.25**	**58.51**	**47.86**	**68.22**	**56.25**	**74.76**	**90.14**	**81.73**
				Populus trichocarpa					
MAKER2*	41.76	42.67	42.21	32.64	38.27	35.23	74.04	71.96	72.99
Funannotate	60.83	54.90	57.71	47.38	54.90	50.86	82.44	79.30	80.84
BRAKER3	**76.99**	**85.60**	**81.07**	**63.07**	**76.49**	**69.13**	**84.56**	**92.94**	**88.55**
				Solanum lycopersicum					
MAKER2*	33.88	18.85	24.22	33.88	18.85	24.22	74.16	45.21	56.17
Funannotate	36.12	34.10	35.08	36.12	34.10	35.08	74.62	65.91	70.00
BRAKER3	**45.90**	**61.67**	**52.63**	**45.90**	**53.49**	**49.41**	**74.87**	**85.25**	**79.72**

Table 6: Sensitivity, specificity, and F1-score for MAKER2, Funannotate, and BRAKER3 using the **close relatives included** protein databases. *The values for MAKER2 may overestimate the realistic performance on new genomes (see main text).

	Gene			Transcript			Exon		
	Sn	Sp	F1	Sn	Sp	F1	Sn	Sp	F1
Funannotate	47.11	44.69	45.87	35.75	44.70	39.73	73.20	74.40	73.79
Funannotate updated	47.11	44.69	45.87	35.75	44.70	39.73	73.20	74.40	73.79
Funannotate –repeats2evm	45.49	45.64	45.56	34.45	45.64	39.27	71.12	77.06	73.97
Funannotate –repeats2evm updated	47.11	44.69	45.87	35.75	44.70	39.73	73.20	74.40	73.79

Table 7: Average sensitivity, specificity, and F1-score for four different predictions generated by Funannotate using the **close relatives included** protein databases for the same species as listed in Table 6. The prediction step of Funannotate was run with and without the option to pass gene predictions of repetitive regions to EVidenceModeler (`--repeats2evm`). The resulting predictions were both post-processed using Funannotate's update protocol, which updates the predicted gene models using RNA-Seq data.

	Arabidopsis thaliana	*Bombus terrestris*	*Caenorhabditis elegans*	*Danio rerio*
BRAKER1	01:23	04:16	01:36	10:28
BRAKER2	06:39	06:27	04:57	19:47
GeneMark-ETP	04:05	02:56	03:36	09:54
BRAKER3	05:37	14:45	06:46	27:08
	Drosophila melanogaster	*Gallus gallus*	*Medicago truncatula*	*Mus musculus*
BRAKER1	01:55	10:12	03:20	19:41
BRAKER2	05:28	14:10	10:59	30:24
GeneMark-ETP	02:37	12:11	07:55	22:13
BRAKER3	06:50	42:44	13:14	64:16
	Parasteatoda tepidariorum	*Populus trichocarpa*	*Solanum lycopersicum*	**Average**
BRAKER1	14:47	04:41	03:47	06:55
BRAKER2	23:16	08:52	09:19	12:45
GeneMark-ETP	10:38	07:51	06:37	08:14
BRAKER3	35:35	11:41	12:06	21:53

Table 8: Runtime of BRAKER1, BRAKER2, GeneMark-ETP, and BRAKER3 for all test species. The runtime is written as hours and minutes. The experiments were run on an HPC node with Intel(R) Xeon(R) CPU E5-2650 v4 @ 2.20GHz using 48 threads.

Species	Runtime (h:m)
Bombus terrestris	4:00
Drosophila melanogaster	2:06
Gallus gallus	13:54
Medicago truncatula	2:48
Populus trichocarpa	3:36

Table 9: Runtime of MAKER2 with *close relatives included* database in MPI mode using LINUX nodes with 96 cores available through the Azure cloud. Training of *ab initio* gene finders and transcriptome assembly are not included in these figures.

	Arabidopsis thaliana	*Bombus terrestris*	*Caenorhabditis elegans*	*Drosophila melanogaster*
Funannotate	05:06	04:59	02:02	02:52
BRAKER3	05:46	14:38	04:13	06:25
	Gallus gallus	*Medicago truncatula*	*Populus trichocarpa*	*Solanum lycopersicum*
Funannotate	41:07	07:11	07:03	09:28
BRAKER3	27:58	07:54	08:19	08:25
	**Average**			
Funannotate	09:59			
BRAKER3	10:27			

Table 10: Runtime of Funannotate and BRAKER3 for the experiments of Table 6 (*close relatives included*). The runtime is written as hours and minutes. The experiments were run on an HPC node with Intel(R) Xeon(R) CPU E5-2650 v4 @ 2.20GHz using 48 threads.

	Gene			Transcript			Exon		
	Sn	Sp	F1	Sn	Sp	F1	Sn	Sp	F1
Arabidopsis thaliana	6.03	18.42	9.09	4.16	16.68	6.66	18.0	50.42	26.53
Caenorhabditis elegans	8.10	12.4	9.80	5.89	11.54	7.80	28.62	50.67	36.58
Danio rerio	3.26	7.17	4.48	1.99	7.06	3.10	15.16	44.12	22.57
Drosophila melanogaster	11.32	23.01	15.17	7.48	21.18	11.06	24.28	53.95	33.49
Medicago truncatula	3.92	16.87	6.36	3.92	15.54	6.26	17.79	48.88	26.09
Populus trichocarpa	7.37	15.34	9.96	5.8	14.75	8.33	26.99	50.07	35.07
Solanum lycopersicum	6.41	12.68	8.52	6.41	10.12	7.85	31.21	43.98	36.51
**Average**	6.63	15.13	9.05	5.09	13.84	7.29	23.15	48.87	30.98

Table 11: Sensitivity, specificity, and F1-score of FINDER for runs using the same input data (genomic sequence + proteins + RNA-Seq) as in the experiments from Table 2. However, FINDER exited with an error for 4 out of the 11 species tested, and we therefore do not report its accuracy for those species.

	Gene			Transcript			Exon		
	Sn	Sp	F1	Sn	Sp	F1	Sn	Sp	F1
Arabidopsis thaliana	78.78	75.18	76.94	54.18	71.77	61.75	83.4	87.70	85.50
Bombus terrestris	67.38	55.01	60.57	46.56	50.28	48.35	84.49	84.17	84.33
Caenorhabditis elegans	70.36	74.46	72.35	51.99	70.34	59.79	84.97	91.99	88.34
Danio rerio	58.06	36.21	44.6	36.29	33.04	34.59	81.74	75.80	78.66
Drosophila melanogaster	80.87	80.71	80.79	54.09	75.66	63.08	83.49	89.93	86.59
Gallus gallus	81.70	61.03	69.87	71.73	57.34	63.73	96.17	89.11	92.51
Medicago truncatula	48.93	46.70	47.79	48.93	43.68	46.16	81.16	69.84	75.08
Mus musculus	79.46	46.23	58.45	79.46	43.42	56.15	94.51	85.48	89.77
Parasteatoda tepidariorum	48.13	26.32	34.03	39.77	24.53	30.34	78.67	65.55	71.51
Populus trichocarpa	75.77	63.60	69.15	60.80	60.97	60.88	87.70	81.99	84.75
Solanum lycopersicum	46.12	37.27	41.23	46.12	34.58	39.52	79.64	66.54	72.50
**Average**	66.87	54.79	59.62	53.63	51.42	51.30	85.09	80.74	82.69

Table 12: Sensitivity, specificity, and F1-score of the AUGUSTUS predictions made as part of the BRAKER3 pipeline. The results correspond to Table 2 – proteins from the same order as the target species were excluded.

Species	SRA ID	#spots	#bases	Date	Species	RNA-Seq lib ID	#spots	#bases	Date
A. tha.	SRR8714016	26,518,474	8,008,579,148	2020-02-27	*B. ter.*	SRR5125126	28,930,495	5,207,489,100	2017-10-30
	SRR8759751	21,388,481	6,458,011,735	2019-04-01		SRR5125123	25,185,030	4,533,305,400	2017-10-30
	SRR4010853	25,658,415	6,465,920,580	2017-05-10		SRR16931591	25,056,341	7,516,902,300	2021-11-12
	SRR7289569	9,222,308	1,391,511,444	2018-12-06		SRR5125133	26,059,038	4,690,626,840	2017-10-30
	SRR12547664	874,772	66,482,672	2021-04-07		SRR5125134	23,745,975	4,274,275,500	2017-10-30
	SRR12076896	21,857,417	6,557,225,100	2021-05-24		SRR8085469	32,768,117	6,553,623,400	2019-03-05
C. ele.	SRR7446944	44,853,824	8,970,764,800	2020-06-01	*D. rer.*	SRR3179613	9,994,443	2,018,877,486	2017-02-10
	ERR2756716	4,785,656	1,445,268,112	2018-08-29		ERR1857957	2,536,494	380,474,100	2017-03-03
	SRR6815567	5,901,194	890,097,086	2018-03-08		SRR9159941	19,527,035	5,858,110,500	2019-12-01
	SRR6474814	34,199,521	8,618,279,292	2018-01-16		SRR9159937	37,156,875	11,147,062,500	2019-12-01
	SRR10238291	47,652,843	4,955,895,672	2019-11-07		ERR958944	10,490,607	1,363,778,910	2015-07-17
						SRR8106574	1,281,189	186,371,148	2020-01-20
D. mel.	SRR19416937	21,377,196	6,413,158,800	2022-05-30	*M. tru.*	SRR3735569	13,021,000	2,630,242,000	2016-07-02
	SRR19416947	22,611,252	6,783,375,600	2022-05-30		SRR2016009	4,289,979	428,997,900	2016-05-12
	SRR19416944	17,754,393	5,326,317,900	2022-05-30		SRR10416790	9,757,388	2,927,216,400	2019-11-23
	SRR19446462	40,819,492	4,163,588,184	2022-05-30		SRR3726824	44,634,145	13,390,243,500	2016-06-28
	SRR19416948	21,257,788	6,377,336,400	2022-05-30		SRR2015998	7,916,226	791,622,600	2016-05-12
						SRR15462191	21,367,770	6,410,331,000	2021-08-15
P. tep.	SRR5458595	94,367,928	18,873,585,600	2017-06-14	*P. tri.*	SRR3019959	32,156,743	4,591,697,381	2016-01-19
	SRR1824488	33,099,205	6,686,039,410	2015-03-03		SRR3019957	44,863,324	6,484,994,933	2016-01-19
	SRR12687629	41,410,278	12,423,083,400	2021-06-22		SRR3019251	29,773,370	4,305,817,693	2016-01-19
	SRR8755634	45,919,505	8,852,599,087	2019-03-20		SRR3019585	36,843,662	5,279,588,065	2016-01-19
	SRR5602551	9,600,853	2,400,213,250	2017-10-16		SRR3019304	41,358,002	5,970,634,746	2016-01-19
	SRR1824489	60,177,256	12,155,805,712	2015-03-03		SRR12671667	17,429,154	5,263,604,508	2020-09-18
G. gal.	SRR5340686	27,956,970	2,851,610,940	2017-03-15	*M. mus.*	SRR5197958	10,577,301	3,184,479,151	2017-08-31
	ERR2113192	20,988,313	3,148,246,950	2017-09-07		SRR3094250	1,568,252	316,786,904	2016-11-14
	ERR2113173	21,562,175	3,234,326,250	2017-09-07		SRR6067921	5,886,768	888,143,922	2018-08-03
	SRR5190436	23,938,529	4,787,705,800	2017-09-06		SRR10115888	44,307,998	8,861,599,600	2020-10-02
	SRR1822373	48,874,648	7,819,943,680	2015-05-05		ERR3005082	8,598	1,289,700	2019-01-02
	SRR5437696	35,378,254	5,306,738,100	2017-09-18		SRR9202226	5,355,073	546,217,446	2019-08-07

Table 13: This table lists all RNA-Seq libraries used for each species in all experiments. It includes the ID of the library at the Sequence Read Archive, the number of spots, number of bases, and publication date.

Table 14: Donor proteins used for each species for the close relative included protein set.

**Species**	**Reference Protein File**
Arabidopsis thaliana	
Arabidopsis lyrata subsp. lyrata	GCF_000004255.2_v.1.0_protein.faa.gz
Arabidopsis thaliana x Arabidopsis arenosa	GCA_019202795.1_ASM1920279v1_protein.faa.gz
Camelina sativa	GCF_000633955.1_Cs_protein.faa.gz
Arabidopsis suecica	GCA_019202805.1_ASM1920280v1_protein.faa.gz
Capsella rubella	GCF_000375325.1_Caprub1_0_protein.faa.gz
Bombus terrestris	
Bombus vancouverensis nearcticus	GCF_011952275.1_Bvanc_JDL1245_protein.faa.gz
Bombus huntii	GCF_024542735.1_iyBomHunt1.1_protein.faa.gz
Bombus affinis	GCF_024516045.1_iyBomAffi1.2_protein.faa.gz
Bombus pyrosoma	GCF_014825855.1_ASM1482585v1_protein.faa.gz
Bombus vosnesenskii	GCF_011952255.1_Bvos_JDL3184-5_v1.1_protein.faa.gz
Bombus bifarius	GCF_011952205.1_Bbif_JDL3187_protein.faa.gz
Bombus impatiens	GCF_000188095.3_BIMP_2.2_protein.faa.gz
Caenorhabditis elegans	
Caenorhabditis auriculariae	GCA_904845305.1_CAUJ_protein.faa.gz
Caenorhabditis bovis	GCA_902829315.1_CBOVIS_v1.1_protein.faa.gz
Caenorhabditis brenneri	GCA_000143925.2_C_brenneri-6.0.1b_protein.faa.gz
Caenorhabditis briggsae	GCF_000004555.2_CB4_protein.faa.gz
Caenorhabditis remanei	GCF_000149515.1_ASM14951v1_protein.faa.gz
Danio rerio	
Cyprinus carpio	GCF_018340385.1_ASM1834038v1_protein.faa.gz
Carassius auratus	GCF_003368295.1_ASM336829v1_protein.faa.gz
Puntigrus tetrazona	GCF_018831695.1_ASM1883169v1_protein.faa.gz
Sinocyclocheilus rhinocerous	GCF_001515625.1_SAMN03320098_v1.1_protein.faa.gz
Sinocyclocheilus anshuiensis	GCF_001515605.1_SAMN03320099.WGS_v1.1_protein.faa.gz
Onychostoma macrolepis	GCA_012432095.1_ASM1243209v1_protein.faa.gz
Carassius gibelio	GCF_023724105.1_carGib1.2-hapl.c_protein.faa.gz
Pimephales promelas	GCF_016745375.1_EPA_FHM_2.0_protein.faa.gz
Labeo rohita	GCF_022985175.1_IGBB_LRoh.1.0_protein.faa.gz
Megalobrama amblycephala	GCF_018812025.1_ASM1881202v1_protein.faa.gz
Sinocyclocheilus grahami	GCF_001515645.1_SAMN03320097.WGS_v1.1_protein.faa.gz
Ctenopharyngodon idella	GCF_019924925.1_HZGC01_protein.faa.gz
Drosophila melanogaster	
Drosophila ananassae	GCF_017639315.1_ASM1763931v2_protein.faa.gz
Drosophila grimshawi	GCF_018153295.1_ASM1815329v1_protein.faa.gz
Drosophila pseudoobscura	GCF_009870125.1_UCI_Dpse_MV25_protein.faa.gz
Drosophila virilis	GCF_003285735.1_DvirRS2_protein.faa.gz
Drosophila willistoni	GCF_018902025.1_UCI_dwil_1.1_protein.faa.gz
Gallus gallus	
Lagopus muta	GCF_023343835.1_bLagMut1_primary_protein.faa.gz
Tympanuchus pallidicinctus	GCF_026119805.1_pur_lepc_1.0_protein.faa.gz
Lagopus leucura	GCF_019238085.1_USGS_WTPT01_protein.faa.gz
Centrocercus urophasianus	GCF_019232065.1_USGS_Curo_1.0_protein.faa.gz
Centrocercus urophasianus	GCF_019232065.1_USGS_Curo_1.0_protein.faa.gz
Coturnix japonica	GCF_001577835.2_Coturnix_japonica_2.1_protein.faa.gz
Meleagris gallopavo	GCF_000146605.3_Turkey_5.1_protein.faa.gz
Medicago truncatula	
Trifolium pratense	GCF_020283565.1_ARS_RC_1.1_protein.faa.gz
Pisum sativum	GCF_024323335.1_CAAS_Psat_ZW6_1.0_protein.faa.gz
Cicer arietinum	GCF_000331145.1_ASM33114v1_protein.faa.gz
Mus musculus	
Arvicanthis niloticus	GCF_011762505.1_mArvNil1.pat.X_protein.faa.gz
Grammomys surdaster	GCF_004785775.1_NIH_TR_1.0_protein.faa.gz
Mastomys coucha	GCF_008632895.1_UCSF_Mcou_1_protein.faa.gz
Mus pahari	GCF_900095145.1_PAHARI_EIJ_v1.1_protein.faa.gz
Apodemus sylvaticus	GCF_947179515.1_mApoSyl1.1_protein.faa.gz
Mus caroli	GCF_900094665.1_CAROLI_EIJ_v1.1_protein.faa.gz
Rattus rattus	GCF_011064425.1_Rrattus_CSIRO_v1_protein.faa.gz
Rattus norvegicus	GCF_015227675.2_mRatBN7.2_protein.faa.gz
Homo sapiens	GCF_000001405.40_GRCh38.p14_protein.faa.gz
Parasteatoda tepidariorum	
Trichonephila inaurata	GCA_019973955.1_Tnin_1.0_protein.faa.gz
Caerostris extrusa	GCA_021605095.1_Cext_1.0_protein.faa.gz
Caerostris darwini	GCA_021605075.1_Cdar_1.0_protein.faa.gz
Oedothorax gibbosus	GCA_019343175.1_Ogib_1.0_protein.faa.gz
Trichonephila clavata	GCA_019973975.1_Tnct_1.0_protein.faa.gz
Trichonephila clavipes	GCA_019973935.1_Tncv_1.0_protein.faa.gz
Araneus ventricosus	GCA_013235015.1_Ave_3.0_protein.faa.gz

Nephila pilipes	GCA_019974015.1_Npil_1.0_protein.faa.gz
Populus trichocarpa	
Populus tomentosa	GCA_018804465.1_PTv2_protein.faa.gz
Populus euphratica	GCF_000495115.1_PopEup_1.0_protein.faa.gz
Populus alba	GCF_005239225.1_ASM523922v1_protein.faa.gz
Populus deltoides	GCA_015852605.2_ASM1585260v2_protein.faa.gz
Solanum lycopersicum	
Solanum stenotomum	GCF_019186545.1_ASM1918654v1_protein.faa.gz
Solanum tuberosum	GCF_000226075.1_SolTub_3.0_protein.faa.gz
Solanum verrucosum	GCF_900185275.1_falcon-dt-bn_protein.faa.gz
Solanum pennellii	GCF_001406875.1_SPENNV200_protein.faa.gz

Species	AUGUSTUS	GeneMark.hmm	SNAP
Arabidopsis thaliana	arabidopsis*	from GeneMark-ES run	A.thaliana.hmm*
Bombus terrestris	bombus_terrestris2*	from GeneMark-EP+ run	trained with genes from ref. annotation
Caenorhabditis elegans	c_elegans_trsk*	from GeneMark-ES run	Ce.hmm*
Drosophila melanogaster	fly*	from GeneMark-ES run	fly*
Gallus gallus	chicken*	medium GC model from GeneMark-ETP run	trained with genes from ref. annotation
Medicago truncatula	trained with genes from ref. annotation	from GeneMark-EP+ run	trained with genes from ref. annotation
Populus trichocarpa	trained with genes from ref. annotation	from GeneMark-EP+ run	trained with genes from ref. annotation
Solanum lycopersicum	tomato*	from GeneMark-EP+ run	trained with genes from ref. annotation

Table 15: Configuration of AUGUSTUS, GeneMark.hmm and SNAP gene models using parameter from the AUGUSTUS and the SNAP distribution packages (marked with *) or with manually training using a training gene set.

1.2 Supplementary Methods

Running BRAKER

BRAKER v3.0.2 was installed from GitHub (`https://github.com/Gaius-Augustus/BRAKER`) and the BRAKER pipelines were run as follows. The GeneMark-ETP results were taken from the BRAKER3 runs:

BRAKER1:

```
braker.pl --genome=genome.softmasked.fasta --threads=48 \
    --rnaseq_sets_dirs=/location/of/local/RNA_Seq/Files \
    --rnaseq_sets_ids=RNA_Seq_IDS
```

BRAKER2:

```
braker.pl --genome=genome.softmasked.fasta --prot_seq=proteins.fa \
     --threads=48
```

BRAKER3:

```
braker.pl --genome=genome.softmasked.fasta --prot_seq=proteins.fa \
    --rnaseq_sets_dirs=/location/of/local/RNA_Seq/Files \
    --rnaseq_sets_ids=RNA_Seq_IDS --threads=48
```

Running Funannotate

Funannotate v1.8.14 was installed using a Singularity container as follows:

```
# only once, to get the singularity container
singularity pull docker://nextgenusfs/funannotate

export GENEMARK_PATH=/path/to/GeneMark-ES-ET-EP_v4.71_lic

species="name of species"
buscoSeedSpecies="name of seed species"
buscodb="name of busco db"
genome="/path/to/genome.fasta.masked"
proteins="/path/to/proteins.fa"
bamfile="/path/to/bamfile.bam"

# calculateGenomeSizeFromFasta.pl adds up the length of all sequences in a fasta
genomeSize=$(perl ~/calculateGenomeSizeFromFasta.pl $genome)
maxIntronLen_f=$(echo "3.6 * sqrt($genomeSize)" | bc -l)
maxIntronLen=$(printf "%.0f" "$maxIntronLen_f")

singularity run funannotate_latest.sif funannotate sort \
    --input $genome \
    --out genome.sorted.fa \
    --simplify
```

We produced four different results for each species, using the option `--repeats2evm` during the prediction step and a recommended update step:

```
mkdir -p fun tmp
# run prediction
singularity run funannotate_latest.sif funannotate predict \
    --input genome.sorted.fa --out fun --species $species \
    --busco_seed_species $buscoSeedSpecies --busco_db $buscodb \
    --organism other --protein_evidence $proteins \
    --rna_bam $bamfile --max_intronlen $maxIntronLen \
    --cpus 72 --tmpdir tmp --no-progress \
    [--repeats2evm]
```

```
# run update step
singularity run funannotate_latest.sif funannotate update \
  --input fun/ --left $readL --right $readR \
  --max_intronlen $maxIntronLen --species $species \
  --memory 50G --cpus 72 --no-progress
```

Running FINDER

FINDER v1.1.0 was installed from GitHub (`https://github.com/sagnikbanerjee15/Finder`) and run as follows:

```
run_finder --protein proteins.fa --framework singularity --output_directory finder --cpu 48 \
    --metadatafile metadata.csv --genome genome.softmasked.fasta --genemark_license gm_key \
    --genemark_path /location/of/GeneMark-ES/ET/EP  --organism_model {VERT,INV,PLANTS,FUNGI}
```

The information in the `metadata.csv` files was manually generated for each species and includes details about the RNA-Seq libraries. The files consist of the following fields:

```
BioProject, SRA Accession, Tissues, Description, Date, Read Length (bp),
Ended, RNA Seq, process, Location
```

Running MAKER2

We ran MAKER2 version 3.01.04 using AUGUSTUS version 3.5.0, GeneMark.hmm version 3.68, SNAP version 2.59.5, Exonerate version 2.2.0, BLAST version 2.14.0, Tandem Repeats Finder version 4.09.

We provided MAKER with a GFF file containing the coordinates of repeats, as well as their predicted repeat types from RepeatMasker. To ensure compatibility with MAKER, we reformatted the RepeatMasker output to a specific GFF format using the following script from `https://github.com/gatech-genemark/BRAKER2-exp.git` and command line:

```
rmasker_out2maker_gff.pl < genome.fasta.out > rmasker4maker.gff
```

This script is available from the repository `https://github.com/gatech-genemark/GeneMark-ETP-exp`.

The default MAKER2 configuration was adjusted with following settings:

```
genome=genome.fasta
est=transcriptome.fasta
protein=proteindb.fasta
model_org=   #empty
rm_gff=repeatmasker.gff
snaphmm=snap.model
gmhmm=genemark.mod
augustus_species=model_name
est2genome=1
protein2genome=1
keep_preds=0
```

Here, `transcriptome.fasta` contained the same transcriptome assemblies that were used with BRAKER3 and constructed with HISAT2 and StringTie2. MAKER2 was then run with:

```
mpiexec.mpich -n 96  maker
```

Preparing protein data

OrthoDB v.11 was partitioned into proteins of species from the clade *Arthropoda*, *Metazoa*, *Vertebrata*, and *Viridiplantae*. The partitioning is available from `https://bioinf.uni-greifswald.de/bioinf/partitioned_odb11/`. Subsequently, two protein sets for each species from Suppl. Table 1 were generated, excluding either only the target species (species-excluded) or all species of the same taxonomic order (order-excluded).
These sets were prepared using the orthodb-clades pipeline, downloaded from GitHub (`https://github.com/tomasbruna/orthodb-clades`).

```
snakemake --cores 48
```

Accuracy evaluation

The accuracy measurements were computed using scripts from the BRAKER suite:

```
compute_accuracies.sh ref_annot.gtf pseudo.gff3 gene_set.gtf gene trans cds
```
